# Surface aggregation patterns of LDL receptors near coated pits III: potential effects of combined retrograde membrane flow-diffusion and a polarized-insertion mechanism

**DOI:** 10.1186/1742-4682-11-23

**Published:** 2014-05-22

**Authors:** Héctor Echavarria-Heras, Cecilia Leal-Ramirez, Oscar Castillo

**Affiliations:** 1 Modeling and Theoretical Analysis Research Group, Centro de Investigación Científica y de Educación Superior de Ensenada, Carretera Ensenada-Tijuana No. 3818, Zona Playitas, C. P. 22869 Ensenada, Baja California, México; 2 Instituto Tecnológico de Tijuana, Tijuana, Baja California, México

**Keywords:** LDL receptor dynamics, Polarized receptor reinsertion, Retrograde-flow model, Mean capture time, Surface-display capping

## Abstract

Although the process of endocytosis of the low density lipoprotein (LDL) macromolecule and its receptor have been the subject of intense experimental research and modeling, there are still conflicting hypotheses and even conflicting data regarding the way receptors are transported to coated pits, the manner by which receptors are inserted before they aggregate in coated pits, and the display of receptors on the cell surface. At first it was considered that LDL receptors in human fibroblasts are inserted at random locations and then transported by diffusion toward coated pits. But experiments have not ruled out the possibility that the true rate of accumulation of LDL receptors in coated pits might be faster than predicted on the basis of pure diffusion and uniform reinsertion over the entire cell surface. It has been claimed that recycled LDL receptors are inserted preferentially in regions where coated pits form, with display occurring predominantly as groups of loosely associated units. Another mechanism that has been proposed by experimental cell biologists which might affect the accumulation of receptors in coated pits is a retrograde membrane flow. This is essentially linked to a polarized receptor insertion mode and also to the capping phenomenon, characterized by the formation of large patches of proteins that passively flow away from the regions of membrane exocytosis. In this contribution we calculate the mean travel time of LDL receptors to coated pits as determined by the ratio of flow strength to diffusion-coefficient, as well as by polarized-receptor insertion. We also project the resulting display of unbound receptors on the cell membrane. We found forms of polarized insertion that could potentially reduce the mean capture time of LDL receptors by coated pits which is controlled by diffusion and uniform insertion. Our results show that, in spite of its efficiency as a possible device for enhancement of the rate of receptor trapping, polarized insertion nevertheless fails to induce the formation of steady-state clusters of receptor on the cell membrane. Moreover, for appropriate values of the flow strength-diffusion ratio, the predicted steady-state distribution of receptors on the surface was found to be consistent with the phenomenon of capping.

## Background

Endocytosis generally refers to the process by which substances are internalized through the cell membrane. Receptor-mediated endocytosis (RME) is a highly specialized kind of endocytosis in which large protein molecules called receptors project from the cell membrane and couple selectively to ligands such as low density lipoproteins (LDL) [1]. Coated pits and their associated receptors have been studied most extensively in cells grown in culture, and the LDL receptor is the one on which a majority of experimental research has been conducted. The ligand-receptor complexes aggregate in specialized cell-membrane formations called coated pits [2-5]. When these close, they form vesicles which then transport the ligand-receptor complexes to the interior of the cell. Once the ligand-receptor bindings are separated, the ligands are degraded at the lysosomes. In some experimental systems, including the LDL system, the receptors are returned to the cell surface for further endocytic cycles, and the ligands are degraded at the lysosomes. Experimental results reveal that RME occurs in virtually all eukaryotic cells except the mature erythrocyte [6] and that it also provides an entrance mechanism for cell nutrients such as low density lipoproteins (LDL) [7], hormones and growth factors [8], serum transport proteins and antibodies [9], as well as toxins and lectins [10] and even viruses [11].

The low density lipoprotein (LDL) particles are cholesterol-transporting macromolecules that are produced in the liver and circulate in the plasma. Membrane reconstitution in human fibroblasts requires the assimilation of cholesterol. The efficiency of this process depends on the rate of endocytosis of the LDL macromolecule and its receptor. It is thought [12] that a severely depleted number of LDL receptors promotes high levels of circulating cholesterol because LDL internalization requires the receptor-binding stage. Deficiencies in the LDL cycle are known to be responsible for the ailment known as familial hypercholesterolemia, which promotes atherosclerosis and the incidence of strokes and coronary disease [13]; this being the reason why so much experimental research has been conducted on the internalization of the LDL receptor. A substantial portion of research aimed at the elucidation of the cholesterol-uptake process has addressed the characterization of the dynamics and display of the LDL receptor. This has produced a large pool of data and a sound conceptual framework which sustains the theoretical exploration of aspects of the dynamics mentioned above.

It is generally thought that the maintenance of receptors on the cell surface is due primarily to receptor recycling rather that to *de novo* synthesis. These data come from experiments using cycloheximide to block protein synthesis. In this experimental setup, it is observed that the number of LDL receptors on the cell surface remain roughly constant for at least six hours [7]. Evidence of receptor internalization and reinsertion in unblocked systems would sustain the assumption that a steady-state concentration of receptors is maintained at the cell surface. The time receptors spend in the interior of the cell is negligible. The basis of this claim is the apparently undetectable pool of receptors inside the cell during endocytosis [14]. The transit time for an LDL receptor from binding on coated pits to reappearance in the membrane, and found it to be on the order of 15 seconds [14]. Based on these ideas, we will abide by the assumption that internalization and recycling of LDL receptors in human fibroblastic cells maintains the surface concentration of LDL at a steady state. Moreover, coated pits include 1% of the cell surface (coated pits include 2% of the cell surface at 4°C [15,16], but when the temperature is raised to 37°C the number of coated pits on the surface is halved [17]). Anderson et al. [17] assert that coated pits tend to be linearly aligned over intracellular fibers. Hence, it can be assumed that the geometrical arrangement of coated pits on the cell surface of human fibroblasts can be reasonably approximated by means of a dilute and ordered system of sinks distributed on a two dimensional surface [18]. Guadorov et al. [19] reported that coated pits tend to form repeatedly at defined sites while other regions are excluded. Therefore, we can assume that coated pits are infinitely long-lived traps distributed in a dilute and ordered form over the cell surface.

It is also known that on cultured human fibroblasts, receptors for certain ligands (e.g. insulin, epidermal growth factor and ∝ - 2-macroglobulin) cluster in coated pits only after exposure to the ligand [20], while receptors for LDL cluster in coated pits independently of ligand binding [7]. This feature of the LDL receptor pathway makes it a particularly attractive candidate for mathematical modeling, since initially we can ignore the details of the ligand-receptor interaction and still study the recycling of the receptor and its interaction with the coated pit. Preliminary mathematical models of RME are aimed at calculating the rate at which diffusing particles (receptors), which have been inserted uniformly all over a certain 2-dimensional space (the cell surface) hit traps (coated pits). This rate is known as the diffusion-limited forward-rate constant [21] and is denoted here by *k*_*d* +_. This rate can be calculated as the flux of particles into a trap, divided by the mean particle concentration [22]. In the two-dimensional case, for a circular sink of radius *a*, *k*_*d* +_ is defined by means of the equation

(1) $$k_{d+}=\frac{2\mathrm{\pi aD}}{\langle C\rangle }\frac{\partial C}{\partial r}|{}_{r=a},$$

where *C*(*r*) is the steady-state radial distribution function of receptors not bound to coated pits, with *D* > 0 their diffusion coefficient and 〈*C*〉 the receptor concentration averaged over all the diffusion space [21], that is,

(2) $$\langle C\rangle =\frac{1}{\pi b^{2}}\int_{0}^{b}2\mathrm{\pi rC}\left(r\right)\mathrm{dr}.$$

The constant *k*_*d* +_ times the number of traps per unit area gives the probability per unit time that a diffusing particle hits the trap. It is linked to the mean time *τ*_*d*_ for a particle to hit a trap (mean capture time) [21] by the relation,

(3) $$\tau_{d}=\frac{1}{k_{d+}\rho },$$

where *ρ* is the number of coated pits distributed per unit area on the cell surface.

If LDL receptors are trapped in times less than, or comparable to the average lifetime of a coated pit (≤5 min), the mean capture time is close to what it would be if the traps were infinitely long-lived. Moreover, since we are dealing with a dilute system of traps, we can follow the method of Berg and Purcell [23] and represent the real multiple-trap problem by means of single coated pit of radius *a*, surrounded by a reference annulus *Ω* of outer radius *b*, i.e.

(4) $$Ω=\left\{\left(r,\theta \right)|\right)0\leq \alpha \leq 2\pi ,\left(a\leq r\leq b\right\},$$

where *b* is calculated through

(5) $$\rho =\frac{1}{\pi b^{2}}$$

Receptors are projected into the reference annulus *Ω* according to an insertion rate function *S*(*r*), and diffuse afterwards until they reach the boundary of the sink at *r* = *a* and are trapped. A steady-state concentration of diffusing particles will be maintained if the number of particles lost to the trap is the same as the number inserted per unit time. Then, if *C*_*ds*_(*r*) denotes the steady-state receptor concentration at a distance *r* from the center of the sink we will have

(6) $$D\nabla^{2}C_{\mathrm{ds}}\left(r\right)+S\left(r\right)=0\;\mathrm{for}\;a\leq r\leq b,$$

so that the inner boundary at *r* = *a* absorbs, i.e.

(7) $$C_{\mathrm{ds}}\left(a\right)=0$$

and the outer boundary at *r* = *b* reflects, i.e.

(8) $$\frac{\partial C_{\mathrm{ds}}}{\partial r}=0\;\mathrm{at}\;r=b,$$

that is, there is no net flux of receptors across the outer boundary of *Ω*. Solving for *C*_*ds*_(*r*) we obtain

(9) $$C_{\mathrm{ds}}\left(r\right)=\int_{a}^{r}\frac{\int_{z}^{b}\mathrm{uS}\left(u\right)\mathrm{du}}{\mathrm{Dz}}\mathrm{dz}.$$

Denoting by means of *C*_*du*_(*r*) the form of *C*_*ds*_(*r*) obtained by setting *S*(*r*) = *S*, and with *S* being a constant, this means that whenever receptors are inserted uniformly over the entire reference annulus *Ω* we obtain

(10) $$C_{\mathrm{du}}\left(r\right)=\frac{b^{2}S}{2D}\ln\left(\frac{r}{a}\right)-\frac{S\left(r^{2}-a^{2}\;\right)\;}{4D}$$

and if *k*_*du* +_ denotes the associated forward rate constant we have,

(11) $$k_{\mathrm{du}+}=\frac{2\mathrm{\pi aD}}{\langle C_{\mathrm{du}}\rangle }\frac{\partial C_{\mathrm{du}}}{\partial r}|{}_{r=a}.$$

Then, for this case equations (1) through (3) yield

(12) $$\tau_{\mathrm{du}}=\frac{b^{4}\ln\left(\frac{b}{a}\right)}{2D\left(b^{2}-a^{2}\right)}-\frac{\left(3b^{2}-a^{2}\right)}{8D}.$$

Goldstein et al. [24] assert that whether traps are ordered or disordered makes only a small difference in the rate at which a trap captures receptors, given that the traps are distributed over the entire surface of the cell in a dilute way. Goldstein et al. [21,25] dealt with two conflicting hypotheses about the coated-pit recycling process. In either case, the coated pit effectively has a finite lifetime. However, they found that the model of equations (6)-(12) obtained under the assumption that sinks have infinite lifetimes give good approximations for *k*_*d* +_ in the experimental system for receptors of low density lipoproteins on human fibroblastic cells. Nevertheless, they also concluded that the experiments do not rule out the possibility that the true rate of accumulation of LDL receptors in coated pits could be faster than predicted on the basis of pure diffusion and uniform reinsertion all over the cell surface. A mechanism that has been proposed by experimental cell biologists which might affect the accumulation of receptors in coated pits is convection. Evidence of a membrane flow comes from the observation that, when the leading edge of a moving cell comes in contact with a small particle, the particle often adheres to the cell surface and is transported backward [26-28]. The motion of such adherent particles is consistent with particles undergoing Brownian motion in the presence of a constant membrane flow [29,30]. It has been hypothesized that this flow originates when membrane components like specific receptors and lipids, which had been internalized from the cell surface, are recycled to the leading edge and contribute to the extension of the cell boundary [29]. Considering that a rapid rate of membrane internalization is observed in endocytosis, a significant amount of recycled membrane should be made available for delivery at the leading edge. This delivery would be restricted to just those molecules that are internalized by coated pits [14,31,32], thus initiating the transport of membrane components away from the leading edge and towards the rear of the cell [26-31]. In addition to the specific delivery of receptors at the leading edge of the cell, their sorting within specific targets along the endocytic pathway have been proposed as components of a mechanism for the control of the internalization of specific ligands [18,33-35]. Generally, eukaryotic cells show the ability to efficiently control the traffic of many types of macromolecules, targeting proteins and lipids for a variety of destinations, based on their types and the current needs of the cell. This traffic control is crucial for the maintenance of cellular structure and function [36,37]. This makes it reasonable to assume that reinsertion of LDL receptors could be arranged in such a way that their trapping rate might be adapted to suit specific metabolic requirements. Moreover, it has been claimed [38] that in giant HeLa cells, receptors are not inserted uniformly into the plasma membrane but rather at the periphery of the cell. Robenek and Hesz [39] claimed that their experiments with LDL particles bound to colloidal gold provided the first clear demonstration of the sequential clustering of receptors near coated pits in plaques or loosely associated groups, and not in the form of widely dispersed individual units. They concluded that this effect is produced when recycled LDL receptors are inserted preferentially in membrane regions where coated pits form. According to Wofsy et al. [40] plaques could be conceptualized as receptor clusters surrounding coated pits. The idea of a retrograde flow is essentially connected to the concept of a polarized reinsertion of receptors. However, neither the influence of this insertion mode on the aggregation rate of LDL receptors in coated pits, nor the resultant steady-state surface aggregation patterns have been formally studied.

## Methods

For the aims of the present research we extend the formal framework presented in Echavarria-Heras et al. [41] for the purpose of including diffusion aided by a retrograde flow, plus polarized receptor insertion as a combination of mechanisms that influences both the mean capture time *τ*_*du*_ and the surface aggregation patterns of unbound LDL receptors. First, we conceptualize a partitioned receptor-insertion mode in which the cell membrane is divided into disjoint regions, each one receiving a fraction of the total number of recycled receptors. In the general form of this mode, different numbers of receptors are projected over the different regions partitioning the cell surface. Polarized insertion, moreover, corresponds to the case in which receptors are recycled into two disjoint regions having abruptly contrasting rates. This partitioned mode can be used to obtain arrangements—which can be seen as special cases—comprising uniform [7], locally uniform, peripheral [38], and plaque forms [39,40], as well as a generalized radially symmetric mode of the form described in [41].

In order to formalize the idea of a partitioned insertion mode, let’s consider the reference annulus *Ω* = {(*r*, *θ*)| *a* ≤ *r* ≤ *b*, - *π* ≤ *θ* ≤ *π*}, letting *I*_*Ω*_ be the number of particles inserted in *Ω*, *m* and *a* being real numbers satisfying 1 ≤ *m* ≤ *b*/*a*, 0 ≤ *α* ≤ *π*, we define the disjoint regions $\Omega_{c}\left(m,\alpha \right),{Ω}_{p}\left(m,\alpha \right)$ and ${Ω}_{q}\left(m,\alpha \right)$ through

(13) $$\Omega_{c}\left(m,\alpha \right)=\left\{\left(r,\theta \right)\left|\;a\leq r\leq b,\right)-\pi +\alpha \leq \theta \leq \pi -\alpha \right\},$$

(14) $$\Omega_{p}\left(m,\alpha \right)=\left\{\left(r,\theta \right)\left|\;\mathrm{ma}\leq r\leq b,\right)\;\pi -\alpha \leq \theta \leq \pi +\alpha \right\},$$

(15) $$\Omega_{q}\left(m,\alpha \right)=\left\{\left(r,\theta \right)\left|\;a\leq r\leq \mathrm{ma},\right)\;\pi -\alpha \leq \theta \leq \pi +\alpha \right\}.$$

Then, we have

(16) $$\Omega =\Omega_{c}\left(m,\alpha \right)\cup \Omega_{p}\left(m,\alpha \right)\cup \Omega_{q}\left(m,\alpha \right).$$

**Figure 1:**
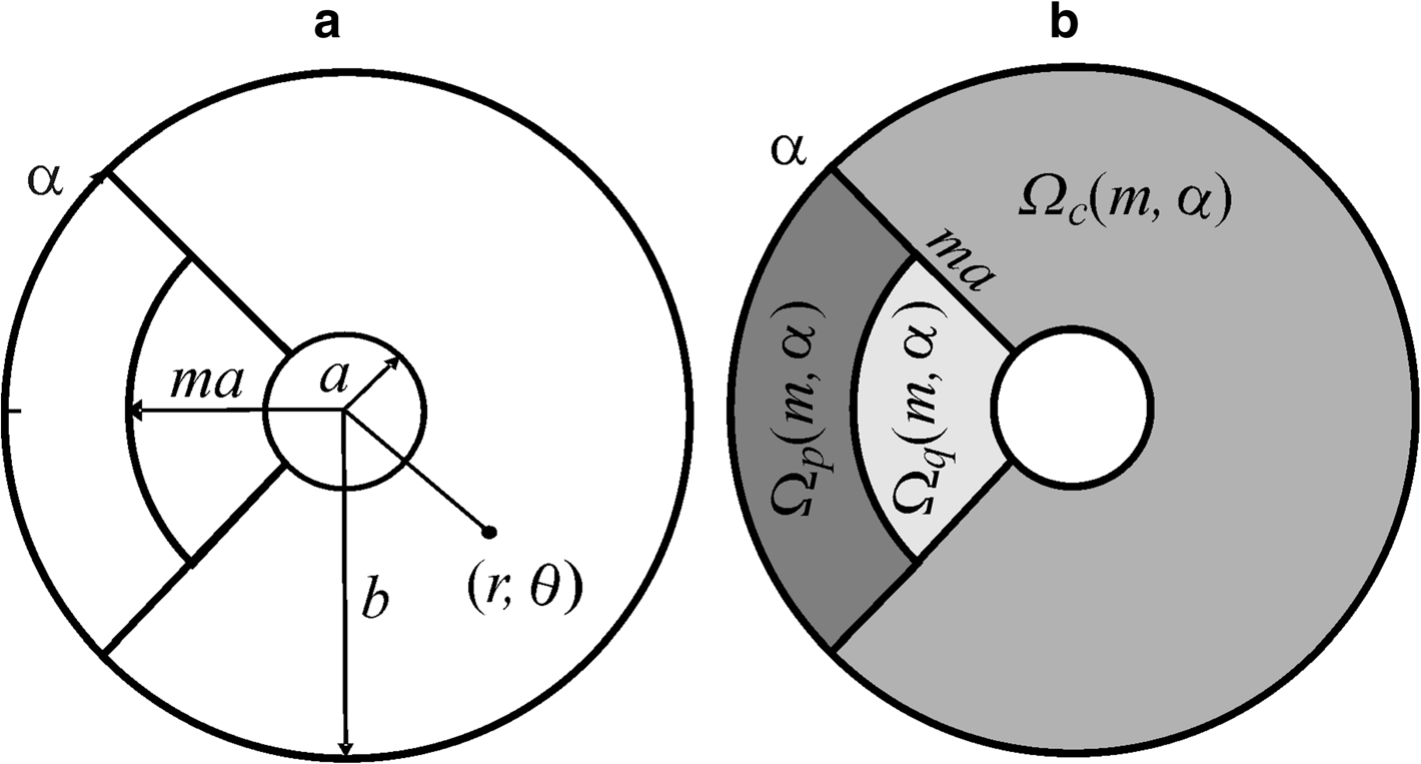
**The geometry of the model. a)** A circular trap of radius *a* (the coated pit) is encircled by an annulus of radius *b* (the reference region *Ω* associated with a coated pit). LDL receptors originally inserted at a point (*r*, *θ*) inside the reference annulus *Ω*, move afterwards by convection and diffusion until they are trapped in coated pits. **b)** Receptor insertion occurs according to a partitioned insertion rate function *S*^*rθ*^(*c*, *p*, *q*, *m*, *α*), which sorts receptors at distinct rates $S_{c}^{\mathrm{r\theta}}\left(m,\alpha \right)$, $S_{p}^{\mathrm{r\theta}}\left(m,\alpha \right)$ and $S_{q}^{\mathrm{r\theta}}\left(m,\alpha \right)$ linked respectively to the disjoint regions *Ω*_*c*_, *Ω*_*p*_ and *Ω*_*q*_.

In order to characterize partitioned receptor insertion, we conceive an insertion rate function *S*^*rθ*^(*c*, *p*, *q*, *m*, *α*)  that distributes receptors over the disjoint regions *Ω*_*c*_(*m*, *α*) *Ω*_*p*_(*m*, *α*), and *Ω*_*q*_(*m*, *α*) (Figure 1) through the respective non-negative and continuous functions $S_{c}^{\mathrm{r\theta}}\left(m,\alpha \right)$, $S_{p}^{\mathrm{r\theta}}\left(m,\alpha \right)$ and $S_{q}^{\mathrm{r\theta}}\left(m,\alpha \right)$. Formally,

(17) $$S^{\mathrm{r\theta}}\left(c,p,q,m,\alpha \right)=\left\{\begin{array}{cc} S_{c}^{\mathrm{r\theta}}\left(m,\alpha \right) & \left(r,\theta \right)\in \Omega_{c}\left(m,\alpha \right)\\ S_{p}^{\mathrm{r\theta}}\left(m,\alpha \right) & \left(r,\theta \right)\in \Omega_{p}\left(m,\alpha \right)\\ S_{q}^{\mathrm{r\theta}}\left(m,\alpha \right) & \left(r,\theta \right)\in \Omega_{q}\left(m,\alpha \right) \end{array}\right),$$

with

(18) $$\iint_{\Omega_{c}}S_{c}^{\mathrm{r\theta}}\left(m,\alpha \right)\mathrm{d\Omega}+\iint_{\Omega_{p}}S_{p}^{\mathrm{r\theta}}\left(m,\alpha \right)\mathrm{d\Omega}+\iint_{\Omega_{q}}S_{q}^{\mathrm{r\theta}}\left(m,\alpha \right)\mathrm{d\Omega}=I_{\Omega }.$$

The formal properties of the partitioned insertion mode are elaborated in Appendix A. Table 1 summarizes the possible forms that *S*^*rθ*^(*c*, *p*, *q*, *m*, *α*) can achieve. Moreover, *S*^*rθ*^(*c*, *p*, *q*, *m*, *α*)  can be handily characterized by using the functions *δ*_*c*_(*m*, *α*), *δ*_*p*_(*m*, *α*) and *δ*_*q*_(*m*, *α*), which respectively stand for the proportion of $I_{Ω}$ that $S_{c}^{\mathrm{r\theta}}\left(m,\alpha \right),$$S_{p}^{\mathrm{r\theta}}\left(m,\alpha \right)$ and $S_{q}^{\mathrm{r\theta}}\left(m,\alpha \right)$ insert over the disjoint regions ${Ω}_{c}\left(m,\alpha \right)$, $\;{Ω}_{p}\left(m,\alpha \right)$ and ${Ω}_{q}\left(m,\alpha \right).$ For *a* and *m* fixed, equations (A4) through (A6) define the proportions *δ*_*c*_(*m*, *α*), *δ*_*p*_(*m*, *α*), and *δ*_*q*_(*m*, *α*). Whenever a pairwise combination of insertion proportions is vanishing, the associated form of the partitioned mode is polarized. Otherwise, the partitioned mode would be uniform all over *Ω* or might be locally uniform, thus sorting receptors at dissimilarly constant rates over ${Ω}_{c}\left(m,\alpha \right),$${Ω}_{p}\left(m,\alpha \right)$ and ${Ω}_{q}\left(m,\alpha \right).$ Although, for ease of presentation, we here partitioned *Ω* into three disjoint regions, the form given by equation (18) could be extended to include a greater number of disjoint regions forming *Ω*.

**Table 1:** **Different forms of the partitioned insertion rate function***S*^*rθ*^(*c*, *p*, *p*, *m*, *α*) : *c***, *p *** **and *q *** **stand for positive constants**

**Mode notation**	**Mode designation**	**Mode defining conditions**	**Eq.**
*S*^*rθ*^(*c*, 0, 0, *b*/*a*, 0)	*c*-uniform	$S_{c}^{\mathrm{r\theta}}\left(\frac{b}{a},0\right)=c,S_{p}^{\mathrm{r\theta}}\left(\frac{b}{a},0\right)=0,S_{q}^{\mathrm{r\theta}}\left(\frac{b}{a},0\right)=0$	A22
*S*^*rθ*^(0, *c*, *c*, *m*, *π*)	*pq*-uniform	$S_{c}^{\mathrm{r\theta}}\left(m,\pi \right)=0,S_{p}^{\mathrm{r\theta}}\left(m,\pi \right)=S_{q}^{\mathrm{r\theta}}\left(m,\pi \right)=c$	A24
*S*^*rθ*^(*c*, *c*, *c*, *m*, *α*)	*cpq*-uniform	$S_{c}^{\mathrm{r\theta}}\left(m,\alpha \right)=S_{p}^{\mathrm{r\theta}}\left(m,\alpha \right)=S_{q}^{\mathrm{r\theta}}\left(m,\alpha \right)=c$	A26
*S*^*rθ*^(*c*, *p*, *q*, *m*, *α*)	*cpq*-locally uniform	$S_{c}^{\mathrm{r\theta}}\left(m,\alpha \right)=c,S_{p}^{\mathrm{r\theta}}\left(m,\alpha \right)=p,S_{q}^{\mathrm{r\theta}}\left(m,\alpha \right)=q$	A28
*S*^*rθ*^(0, *p*, *q*, *m*, *π*)	*pq*-locally uniform	$S_{c}^{\mathrm{r\theta}}\left(m,\pi \right)=0,S_{p}^{\mathrm{r\theta}}\left(m,\pi \right)=p,S_{q}^{\mathrm{r\theta}}\left(m,\pi \right)=q$	A30
*S*^*rθ*^(0, 0, *q*, *m*, *π*)	*q*-plaque form	$S_{c}^{\mathrm{r\theta}}\left(m,\pi \right)=0,S_{p}^{\mathrm{r\theta}}\left(m,\pi \right)=0,S_{q}^{\mathrm{r\theta}}\left(m,\pi \right)=q$	A32
*S*^*rθ*^(0, *p*, 0, *m*, *π*)	*p*-peripherial	$S_{c}^{\mathrm{r\theta}}\left(m,\pi \right)=0,S_{p}^{\mathrm{r\theta}}\left(m,\pi \right)=p,S_{q}^{\mathrm{r\theta}}\left(m,\pi \right)=0$	A34
*S*^*rθ*^(0, *p*, 0, *m*, *α*)	*p*-polarized	$S_{c}^{\mathrm{r\theta}}\left(m,\alpha \right)=0,S_{p}^{\mathrm{r\theta}}\left(m,\alpha \right)=p,S_{q}^{\mathrm{r\theta}}\left(m,\alpha \right)=0$	A36
*S*^*rθ*^(0, *p*, *q*, *m*, *α*)	*pq*-polarized	$S_{c}^{\mathrm{r\theta}}\left(m,\alpha \right)=0,S_{p}^{\mathrm{r\theta}}\left(m,\alpha \right)=p,S_{q}^{\mathrm{r\theta}}\left(m,\alpha \right)=q$	A38
*S*^*rθ*^(0, 0, *q*, *m*, *α*)	*q*-polarized	$S_{c}^{\mathrm{r\theta}}\left(m,\alpha \right)=0,S_{p}^{\mathrm{r\theta}}\left(m,\alpha \right)=0,S_{q}^{\mathrm{r\theta}}\left(m,\alpha \right)=q$	A40

Let *C*_*λmα*_(*r*, *θ*) denote the steady-state concentration of receptors in *Ω*. In Appendix B we review the formal steps which establish that if receptors are inserted in *Ω* in accordance with the insertion rate function *S*^*rθ*^(*c*, *p*, *q*, *m*, *α*) and afterwards move by diffusion aided by a retrograde membrane flow, then if *λ* = *v*/2*D*, where *v* is the strength of the retrograde flow and *D* stands for the diffusion coefficient (cf. Eq. B1), we will have

(19) $$C_{\mathrm{\lambda m\alpha}}\left(r,\theta \right)=G_{\mathrm{\lambda m\alpha}}\left(r,\theta \right){e^{\lambda }}^{r\cos\theta },$$

where *G*_*λmα*_(*r*, *θ*)  is the solution to the boundary-value problem,

(20) $$\nabla^{2}G_{\mathrm{\lambda m\alpha}}\left(r,\theta \right)-\lambda^{2}G_{\mathrm{\lambda m\alpha}}\left(r,\theta \right)=-\frac{S^{\mathrm{r\theta}}\left(c,p,q,m,\alpha \right)\;}{D}e^{-\mathrm{\lambda b}\cos\theta },$$

(21) $$G_{\mathrm{\lambda m\alpha}}\left(a,\theta \right)=0,$$

(22) $$G_{\mathrm{\lambda m\alpha}}\left(r,\theta \right)=G_{\mathrm{\lambda m\alpha}}\left(r,-\theta \right),$$

(23) $$G_{\mathrm{\lambda m\alpha}}\left(r,\theta \right)\mathrm{periodic}\;\mathrm{in}\;\theta ,$$

(24) $$\int_{-\pi }^{\pi }\left(\frac{\partial G_{\mathrm{\lambda m\alpha}}\left(r,\theta \right)}{\partial r}|{r=b}^{-\lambda \cos\;\theta \;G_{\mathrm{\lambda m\alpha}}\left(b,\theta \right)}\right)e^{-\mathrm{\lambda b}\cos\theta }\mathrm{d\theta}=0.$$

The associated forward rate constant is denoted by means of *k*_*mα* +_ and is given by

(25) $$k_{\mathrm{\lambda m\alpha}+}=\frac{\pi b^{2}\int_{a}^{b}\int_{-\pi }^{\pi }S^{\mathrm{r\theta}}\left(c,p,q,m,\alpha \right)\;\mathrm{rdrd\theta}}{\int_{a}^{b}\int_{-\pi }^{\pi }C_{\mathrm{\lambda m\alpha}}\left(r,\theta \right)\mathrm{rdrd\theta}},$$

and the associated mean capture time *τ*_*λmα*_ is given by

(26) $$\tau_{\mathrm{\lambda m\alpha}}=\frac{\int_{a}^{b}\int_{-\pi }^{\pi }G_{\mathrm{\lambda m\alpha}}\left(r,\theta \right)e^{\mathrm{\lambda r}\cos\theta }\mathrm{rdrd\theta}}{\int_{a}^{b}\int_{-\pi }^{\pi }S^{\mathrm{r\theta}}\left(c,p,q,m,\alpha \right)\;\mathrm{rdrd\theta}}.$$

For particular characterizations of *S*^*rθ*^(*c*, *p*, *q*, *m*, *α*), the resulting patterns of receptors can be simulated. These representations can be obtained by means of the computer graphics technique of ray tracing [42] using grey tones corresponding to the values of *C*_*λmα*_(*r*, *θ*).

## Results and discussion

Anderson et al. [7] proposed that LDL receptors in human fibroblasts are inserted at random locations and are transported by diffusion toward coated pits, and Goldstein et al. [21,25] have studied theoretical aspects of the dynamics of the LDL receptor on the cell surface. One of the questions addressed in these models was whether or not the random insertion of LDL receptors into the plasma membrane, followed by pure diffusion and using the measured diffusion coefficient for LDL receptors, could give a rapid enough aggregation of receptors in coated pits to account for the observed rate of removal of LDL from the cell surface. Since an experimentally determined lower bound for the forward rate constant *k*_*du* +_ is (2.3 × 10^- 10^ ± 1.6) × 10^- 10^*cm*^2^/*s* and the value obtained from their model is 1.9 × 10^- 10^*cm*^2^/*s*, the answer to that question is that the hypothesis of random insertion and pure diffusion of LDL receptors to coated pits is consistent with experimental observations but just barely. Therefore, even though the diffusion-limited forward-rate constant *k*_*du* +_ calculated by these authors is consistent with available experimental results on the rate of LDL internalization, these do not rule out the possibility that the true value of the mean capture time of receptors is actually smaller than *τ*_*du*_, the value predicted on the basis of random insertion of receptors followed by pure diffusion to coated pits. Indeed, if LDL receptors on human fibroblasts are not inserted uniformly into the plasma membrane, but are inserted preferentially into specialized regions where coated pits form, and if the resulting reduction in the time required for LDL receptors to diffuse to coated pits was significant, the conclusion that diffusion of LDL receptors to coated pits is the limiting step in the interaction of cell surface LDL receptors with coated pits could be wrong. Also, diffusion could be aided by a more active transport in order to increase the rate of accumulation of receptors in coated pits. In particular, we have studied here the effects of a polarized insertion of receptors as well as those related to a transport mechanism set by diffusion aided by a retrograde membrane flow.

Experimentally determined values for coated pit radius *a* and the outer radius *b* of the reference region *Ω* are respectively *a* = 0.10 ± 0.05 *μm*[43] and *b* = 1.0 ± 0.2 *μm*; these were obtained by using equation (5) with the calculated value for the suitable density of coated pits *ρ* = 0.31 ± 0.09/*μm*^2^[16]. In order to typify values of the fundamental ratio *λ* = *v*/2*D* (cf. Eq. B1) we will use a low reference value *v* = *v*_0_ for the strength *v* of the retrograde membrane flow. The value *v*_0_ = 9.6 × 10^- 7^ *cm*/*min* represents the maximum of a hypothetical local flow that originates when coated pits invaginate to internalize the trapped ligand-receptor complexes [44]. As a second reference value for the strength *v* of the retrograde flow we may take *v*_1_ = 10^- 4^ *cm*/*min*. This value gives the approximate rate at which objects on the surface of fibroblasts are swept backwards [26,14]. Since *v*_1_ is approximately equal to 100*v*_0_, this retrograde membrane-flow rate would sustain an extremely fast convective transport in comparison with one having a strength *v*_0_. In order to characterize a variation range for the diffusion coefficient *D*, we will consider a normal process for the LDL receptor. This is associated with a diffusion coefficient value of *D*_0_ = (2.7 ± 0.09) × 10^- 9^*cm*2/*min*, which was determined by Barak and Webb [45] using fluorescence photo bleaching recovery (FBR) in an experimental arrangement involving LDL receptors diffusing on the surface of human fibroblast cells. We can also contemplate a fast diffusion process corresponding to *D*_1_ = 8.1 × 10^- 8^ *cm*2/*min*, this being the average of the values defining the observed range of variation for the diffusion coefficient of the dil (3)- LDL receptor complex on blebs induced in the cell membrane. And we could also consider an extreme reference value of *D*_*ext*_ = 6 × 10^- 7^*cm*^2^/*min*, that is *D*_*ext*_ = 7.40*D*_1_.

The phenomenon of capping is characterized by the formation of large patches of proteins that passively flow away from the regions of membrane exocytosis. Individual proteins can overcome this convective transport when they have diffusion coefficients (*D*) of about *D*_*ext*_[14]. Moreover, a value for *D* of *D*_*ext*_/6 is comparable to the largest estimation for the diffusion coefficient of the dil (3)- LDL receptor complex on blebs [45]. For the parameter values of the LDL system on human fibroblasts, the calculated value for *τ*_*du*_, the mean capture time when receptors move by pure diffusion and are inserted uniformly over the entire cell surface, is *τ*_*du*_ = 2.92 *min* (cf. Eq. (11)).

**Figure 2:**
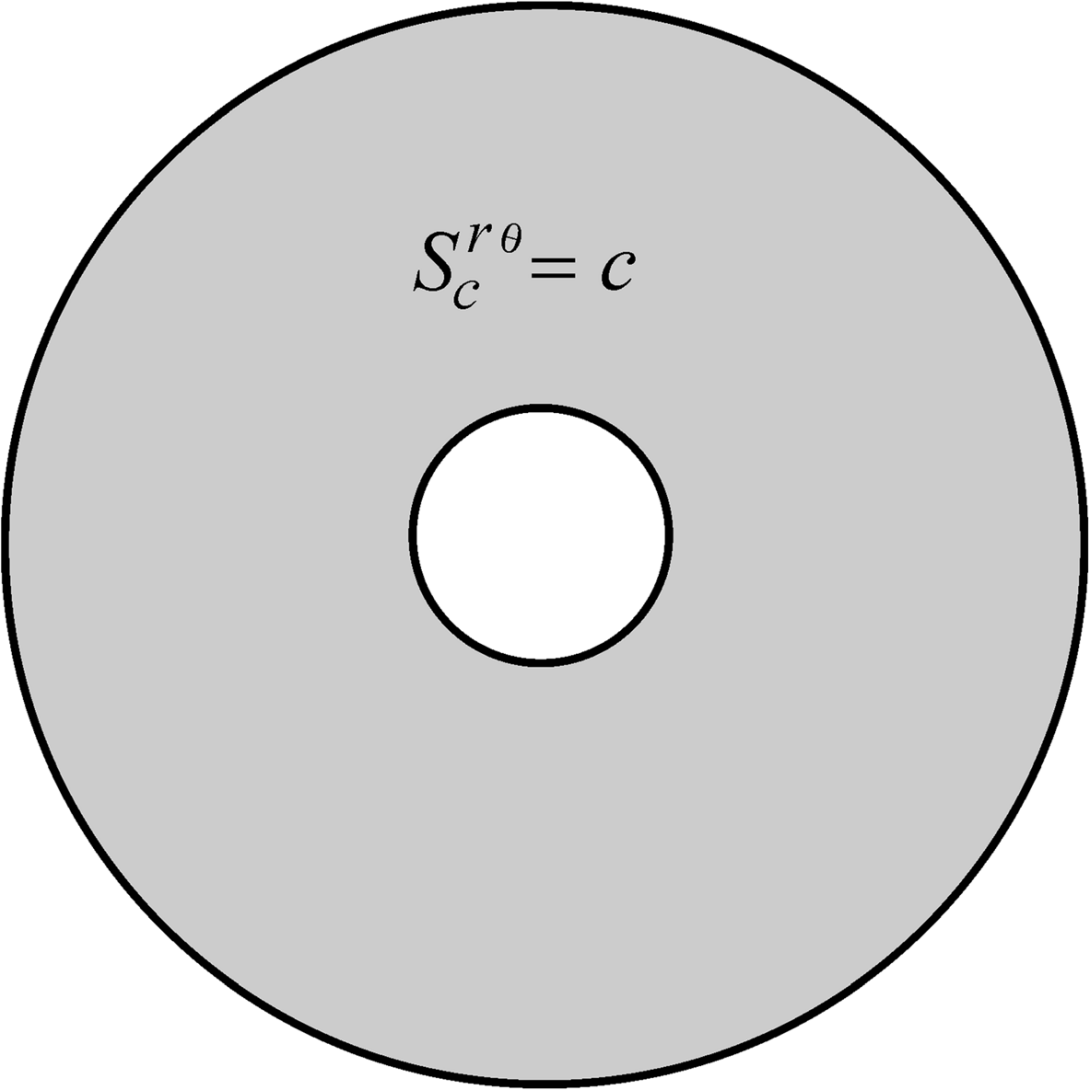
**The *c*-uniform insertion mode.** A receptor insertion mechanism symbolized here by means of *S*^*rθ*^(*c*, 0, 0, *b*/*a*, 0), which is obtained by setting $\;S_{p}^{\mathrm{r\theta}}\left(b/a,0\right)=S_{q}^{\mathrm{r\theta}}\left(m,\alpha \right)=0$ and $S_{c}^{\mathrm{r\theta}}\left(b/a,0\right)=c$, with *c* a positive constant.

**Figure 3:**
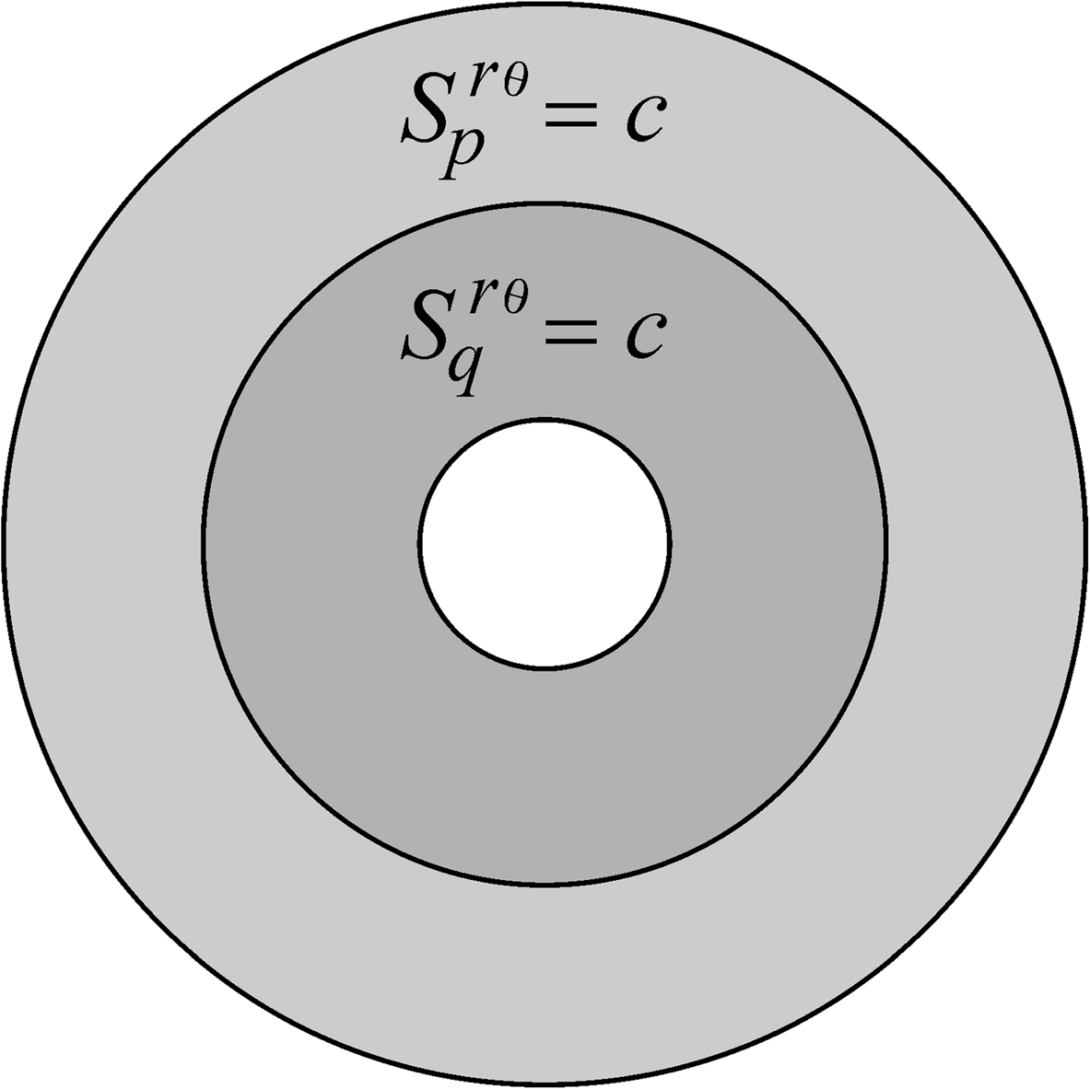
**The *pq*-uniform insertion mode.** This is denoted by means of the symbol *S*^*rθ*^(0, *c*, *c*, *m*, *π*), and obtained by setting the conditions $S_{c}^{\mathrm{r\theta}}\left(m,\pi \right)=0$ and $S_{p}^{\mathrm{r\theta}}\left(m,\pi \right)=S_{q}^{\mathrm{r\theta}}\left(m,\pi \right)=c$, with *c* a positive constant.

**Figure 4:**
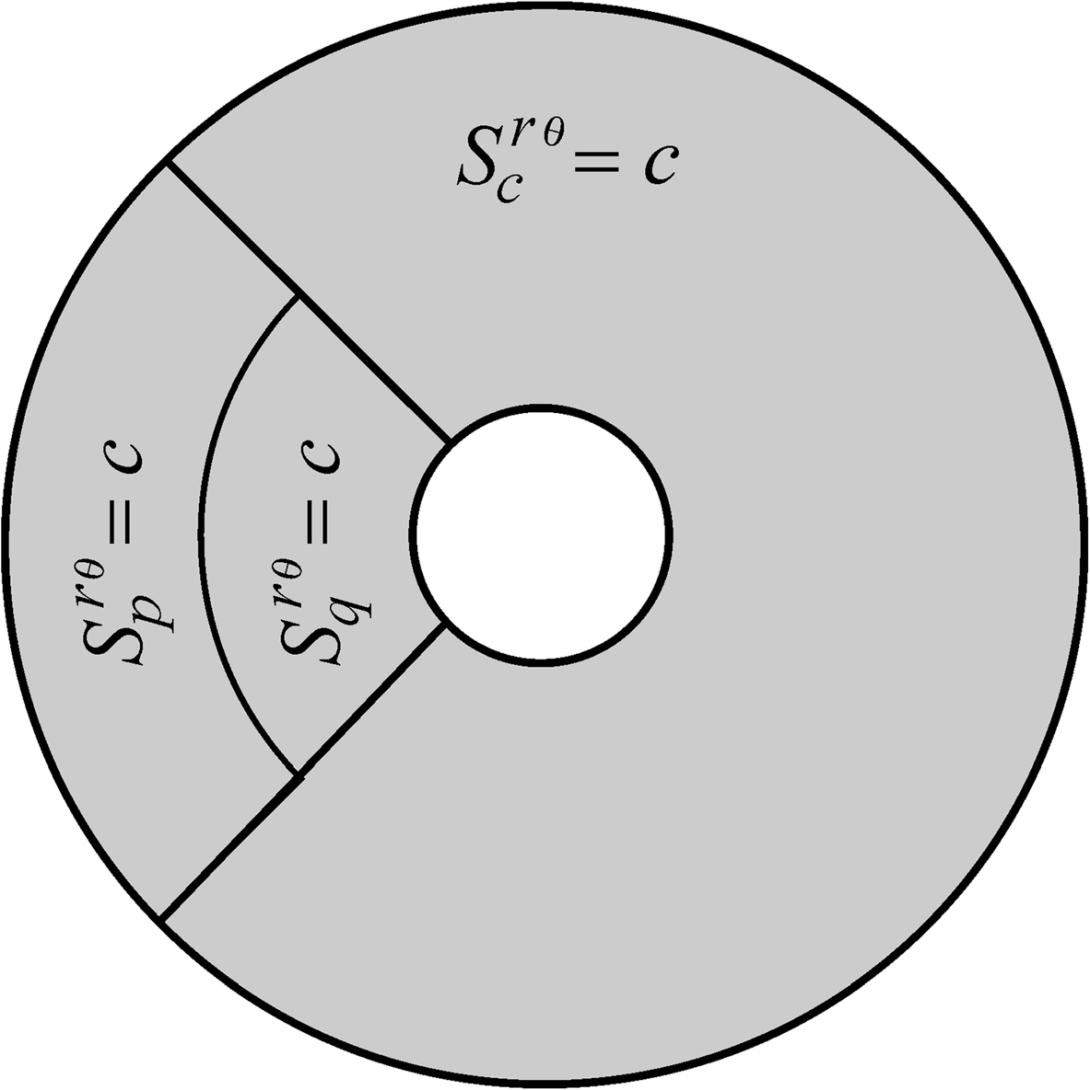
**The *cpq*-uniform insertion mode.** A device symbolized here using *S*^*rθ*^(*c*, *c*, *c*, *m*, *α*) and is obtained by setting $S_{c}^{\mathrm{r\theta}}\left(m,\alpha \right)=S_{p}^{\mathrm{r\theta}}\left(m,\alpha \right)=S_{q}^{\mathrm{r\theta}}\left(m,\alpha \right)=c$, with *c* a positive constant.

In the present setting, a uniform insertion-rate function can be acquired by, for instance taking *α* = 0, *m* = *b*/*a* and $S_{c}^{\mathrm{r\theta}}\left(m,0\right)=c$, where *c* is a positive constant and $S_{p}^{\mathrm{r\theta}}\left(m,0\right)=S_{q}^{\mathrm{r\theta}}\left(m,0\right)=0$. This representation is known here as a *c*-uniform insertion mode (Figure 2 and Eq. A22). A second description of a uniform insertion mode is what we call a *pq*-uniform rate function, in which receptors are inserted solely over the regions *Ω*_*p*_(*m*, *π*) and *Ω*_*q*_(*m*, *π*); this is realized by setting $S_{p}^{\mathrm{r\theta}}\left(m,\pi \right)=S_{q}^{\mathrm{r\theta}}\left(m,\pi \right)=c$, where *c* is a positive constant (Figure 3 and Eq. A24). Finally, a description that we refer to as *cpq*-uniform insertion is achieved by sorting receptors over the three disjoint regions *Ω*_*c*_(*m*, *α*), *Ω*_*p*_(*m*, *α*), *Ω*_*q*_(*m*, *α*)  with $S_{c}^{\mathrm{r\theta}}\left(m,\alpha \right)=S_{p}^{\mathrm{r\theta}}\left(m,\alpha \right)=S_{q}^{\mathrm{r\theta}}\left(m,\alpha \right)=c$, where *c* is a positive constant (Figure 4 and Eq. A26). Consistently, for the case where no convective flow is present (*v* = 0) and receptors diffuse normally (*D* = *D*_0_), all these representations indistinctively yield *τ*_*λmα*_ = *τ*_*du*_.

**Figure 5:**
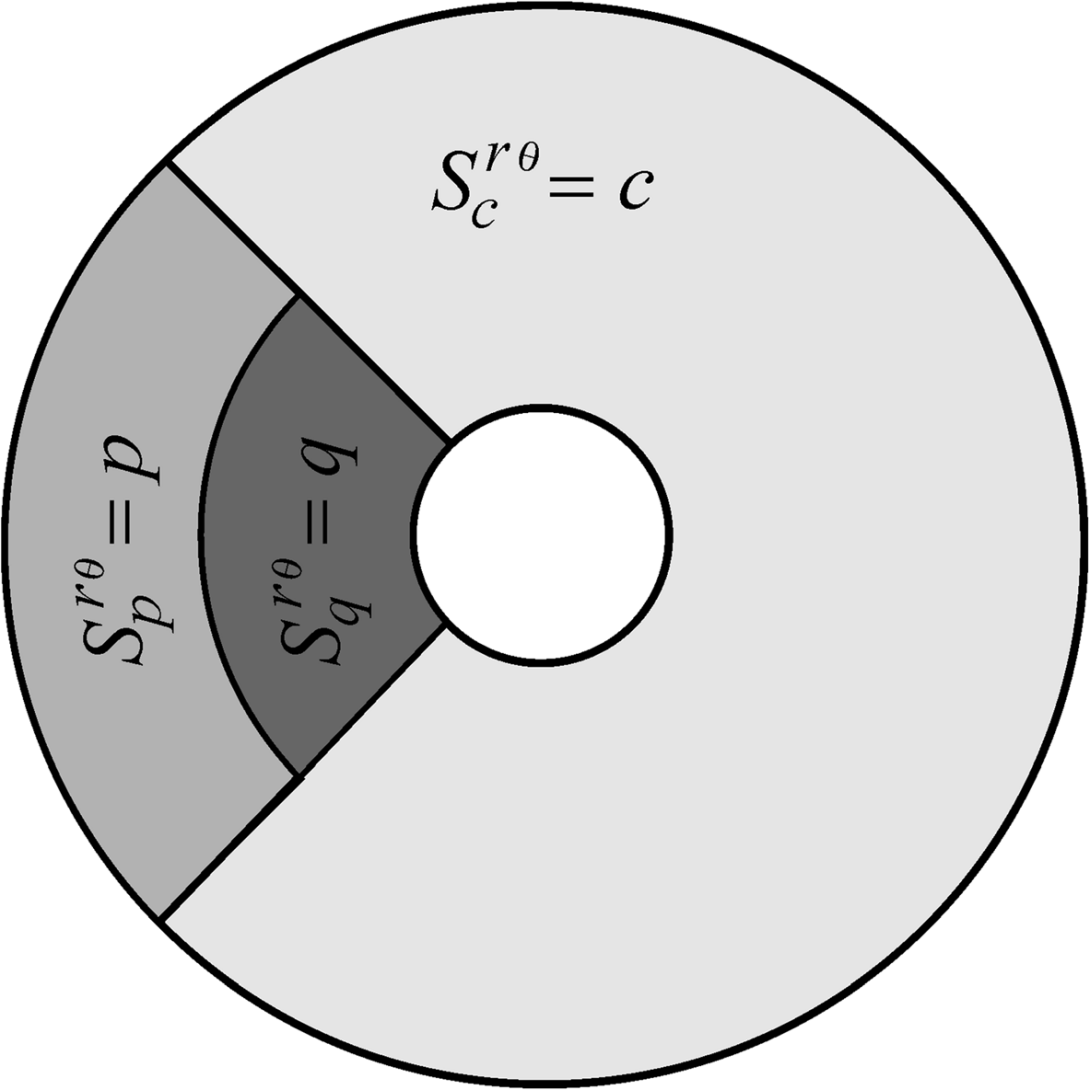
**The *cpq*-locally uniform insertion mode.** This insertion rate function is labeled by means of *S*^*rθ*^(*c*, *p*, *q*, *m*, *α*), it is linked to the conditions $S_{c}^{\mathrm{r\theta}}\left(m,\alpha \right)=c$, $S_{p}^{\mathrm{r\theta}}\left(m,\alpha \right)=p$, and $S_{q}^{\mathrm{r\theta}}\left(m,\alpha \right)=q$, with *c*, *p* and *q* positive constants.

**Figure 6:**
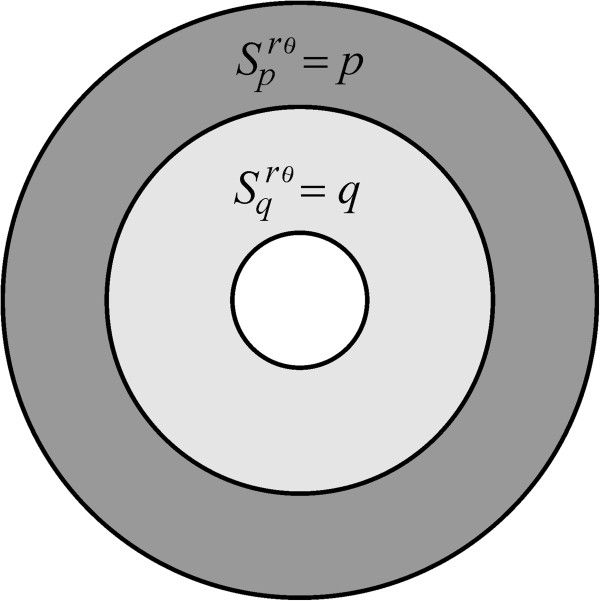
**The *pq*-locally uniform insertion mode.** A receptor insertion mechanism which is denoted by means of *S*^*rθ*^(0, *p*, *q*, *m*, *π*). It is radially symmetric over each one of the regions *Ω*_*p*_ and *Ω*_*q*_. This form is associated with the case $S_{c}^{\mathrm{r\theta}}\left(m,\pi \right)=0$, $S_{p}^{\mathrm{r\theta}}\left(m,\pi \right)=p$, and $S_{q}^{\mathrm{r\theta}}\left(m,\pi \right)=q$, with *p* and *q* positive constants.

**Figure 7:**
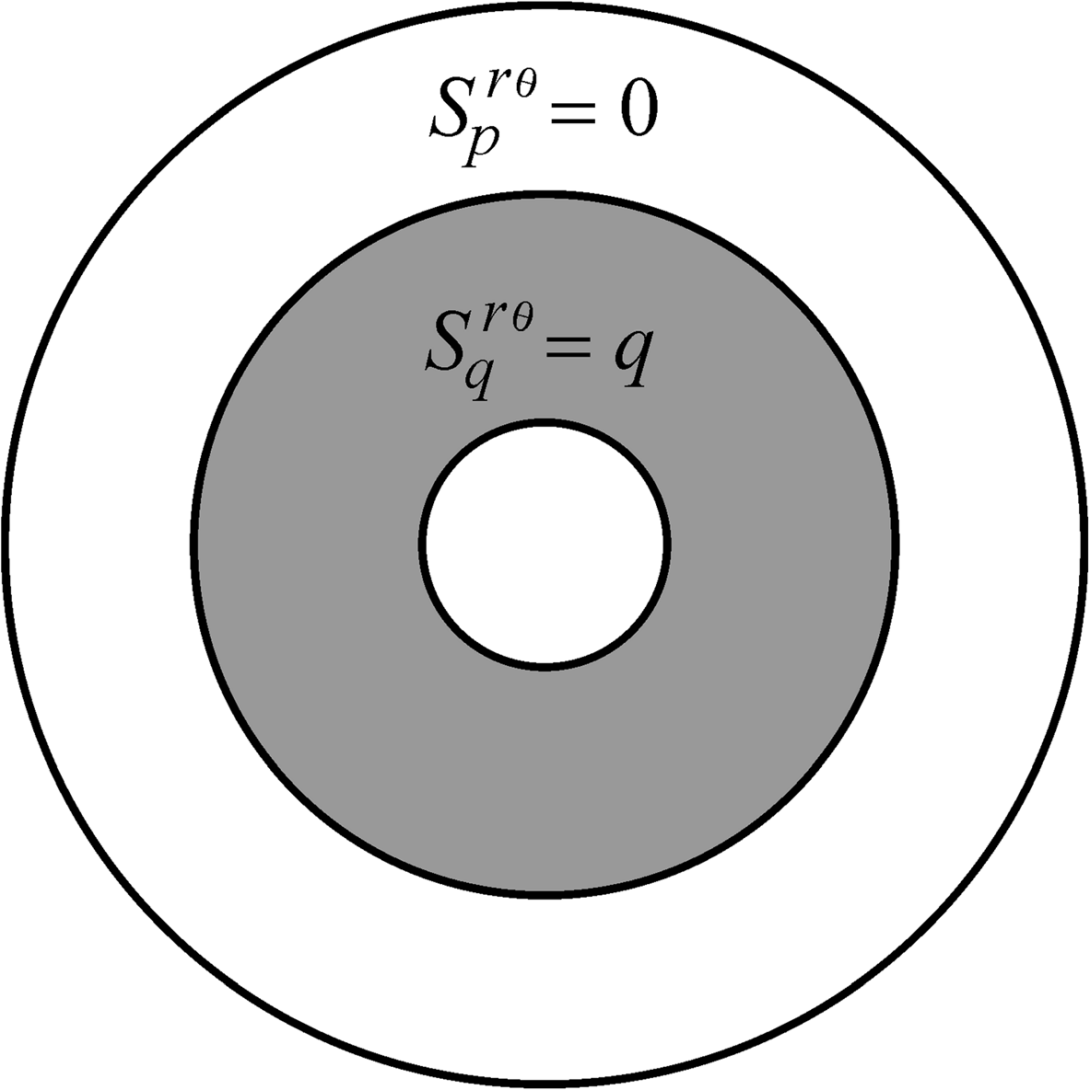
**The *q*-plaque form insertion mode.** This mode is denoted here by means of the symbol *S*^*rθ*^(0, 0, *q*, *m*, *π*). It is radially symmetric and polarized and associated with the conditions $S_{c}^{\mathrm{r\theta}}\left(m,\pi \right)=0$, $S_{p}^{\mathrm{r\theta}}\left(m,\pi \right)=0$, and $S_{q}^{\mathrm{r\theta}}\left(m,\pi \right)=q$, with *q* a positive constant.

**Figure 8:**
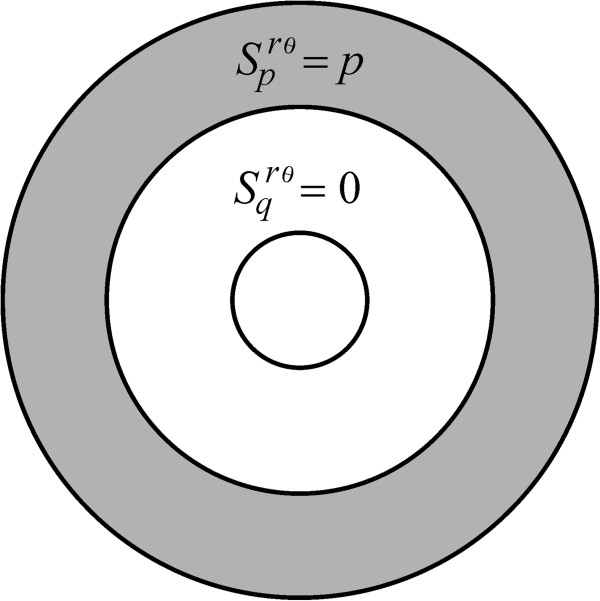
**The *p*-peripheral insertion mode.** An insertion form denoted here by means of *S*^*rθ*^(0, *p*, 0, *m*, *π*). It is a radially symmetric and polarized and linked to the case $S_{c}^{\mathrm{r\theta}}\left(m,\pi \right)=0$, $S_{p}^{\mathrm{r\theta}}\left(m,\pi \right)=p$, and $S_{q}^{\mathrm{r\theta}}\left(m,\pi \right)=0$, with *p* a positive constant.

For the case in which convective transport is moderate (*v* = *v*_0_) and receptors diffuse normally (*D* = *D*_0_), that is, the flow strength to diffusion ratio *λ* has a value *λ* = *v*_0_/2*D*_0_, calculating *τ*_*λmα*_ as given by equation (B27) for *S*^*rθ*^(*c*, *p*, *q*, *m*, *α*) where insertion is uniform over all of *Ω*, we obtained *τ*_*λmα*_ = 1.003 *τ*_*du*_. Hence, the present model predicts that if LDL receptors diffuse normally, a retrograde membrane flow having strength comparable to *v*_0_ would have an insignificant effect on *τ*_*du*_. But for that same convection-to-diffusion ratio, we found that a locally uniform insertion mode can prompt greater receptor trapping rates than those linked to uniform insertion all over *Ω*. But now suppose that receptor insertion takes place over the regions *Ω*_*c*_(*m*, *α*), *Ω*_*p*_(*m*, *α*) and *Ω*_*q*_(*m*, *α*), at different constant rates $S_{c}^{\mathrm{r\theta}}\left(m,\alpha \right)=c,S_{p}^{\mathrm{r\theta}}\left(m,\alpha \right)=p$ and $S_{q}^{\mathrm{r\theta}}\left(m,\alpha \right)=q$; this results in what we term here a *cpq*-locally uniform insertion mode (Figure 5 and Eq. A28). Then, if for instance we choose *m* = 1.5, *α* = *π*/2, *δ*_*c*_(*m*, *α*) = 0.10, *δ*_*p*_(*m*, *α*) = 0.30 and *δ*_*q*_(*m*, *α*) = 0.60, the model yields *τ*_*λmα*_ = 0.49*τ*_*du*_, which amounts to an important reduction of *τ*_*du*_. For *α* = *π* we obtain what we label here as a *pq*-locally uniform insertion form (Figure 6 and Eq. 30) denoted by the symbol *S*^*rθ*^(0, *p*, *q*, *m*, *π*). Receptors are inserted in a radially symmetric manner over each of the regions *Ω*_*q*_(*m*, *π*) and *Ω*_*p*_(*m*, *π*), but through different constant rates $S_{p}^{\mathrm{r\theta}}\left(m,\alpha \right)=p$ and $S_{q}^{\mathrm{r\theta}}\left(m,\alpha \right)=q.$ For *m* = 2.0, *δ*_*p*_(*m*, *π*) = 0.20 and *δ*_*q*_(*m*, *π*) = 0.80, the model yields *τ*_*λmα*_ = 0.41*τ*_*du*_. Now, since for a *pq*-locally uniform insertion form we have *δ*_*p*_(*m*, *π*) + *δ*_*q*_(*m*, *π*) = 1, then letting *δ*_*p*_(*m*, *π*) approach zero, receptor insertion will be gradually accommodated within the region *Ω*_*q*_(*m*, *α*)  so that eventually, when *δ*_*p*_(*m*, *π*) vanishes, we will obtain what we identify as a *q*-plaque form insertion mode denoted by means of the symbol *S*^*rθ*^(0, 0, *q*, *m*, *π*) (Figure 7 and Eq. A32). This is actually the plaque-form insertion mechanism envisioned by Wofsy et al. [40] for modeling preferential insertion as conceived by Robeneck and Hesz [39]. For *λ* = *v*_0_/*D*_0_, and *m* = 2.0, the mode *S*^*rθ*^(0, 0, *q*, *m*, *π*) yields *τ*_*λmα*_ = 0.26*τ*_*du*_, again a noticeable reduction in *τ*_*du*_. This mode is radially symmetric and can be already considered as a form of polarized insertion. Similarly, if we initially chose a locally uniform mode *S*^*rθ*^(0, *p*, *q*, *m*, *π*), then letting *δ*_*q*_(*m*, *π*) approach zero we will force receptors to be mainly sorted over the *Ω*_*p*_(*m*, *π*), region and in due course when *δ*_*q*_(*m*, *π*) vanishes will produce what we call a *p*-peripheral insertion mode symbolized by means of *S*^*rθ*^(0, *p*, 0, *m*, *π*) (Figure 8 and Eq. A34). This insertion rate function is a peripheral form of polarized insertion; receptors are inserted in an annulus contiguous to the outer boundary of *Ω*. For *λ* = *v*_0_/*D*_0_, peripheral insertion yields *τ*_*λmα*_ ≥ 1.003 *τ*_*du*_ with the lower bound of 1.003 *τ*_*du*_ attained in the limiting case when *m* approaches one. Hence, if enhancement of LDL receptor trapping rate is required, a peripheral insertion mode for *m* > 1 turns out to be an inefficient mechanism. For instance taking *λ* = *v*_0_/2*D*_0_, and *m* = 9.7 we obtain *τ*_*λmα*_ = 1.15*τ*_*du*_.

**Figure 9:**
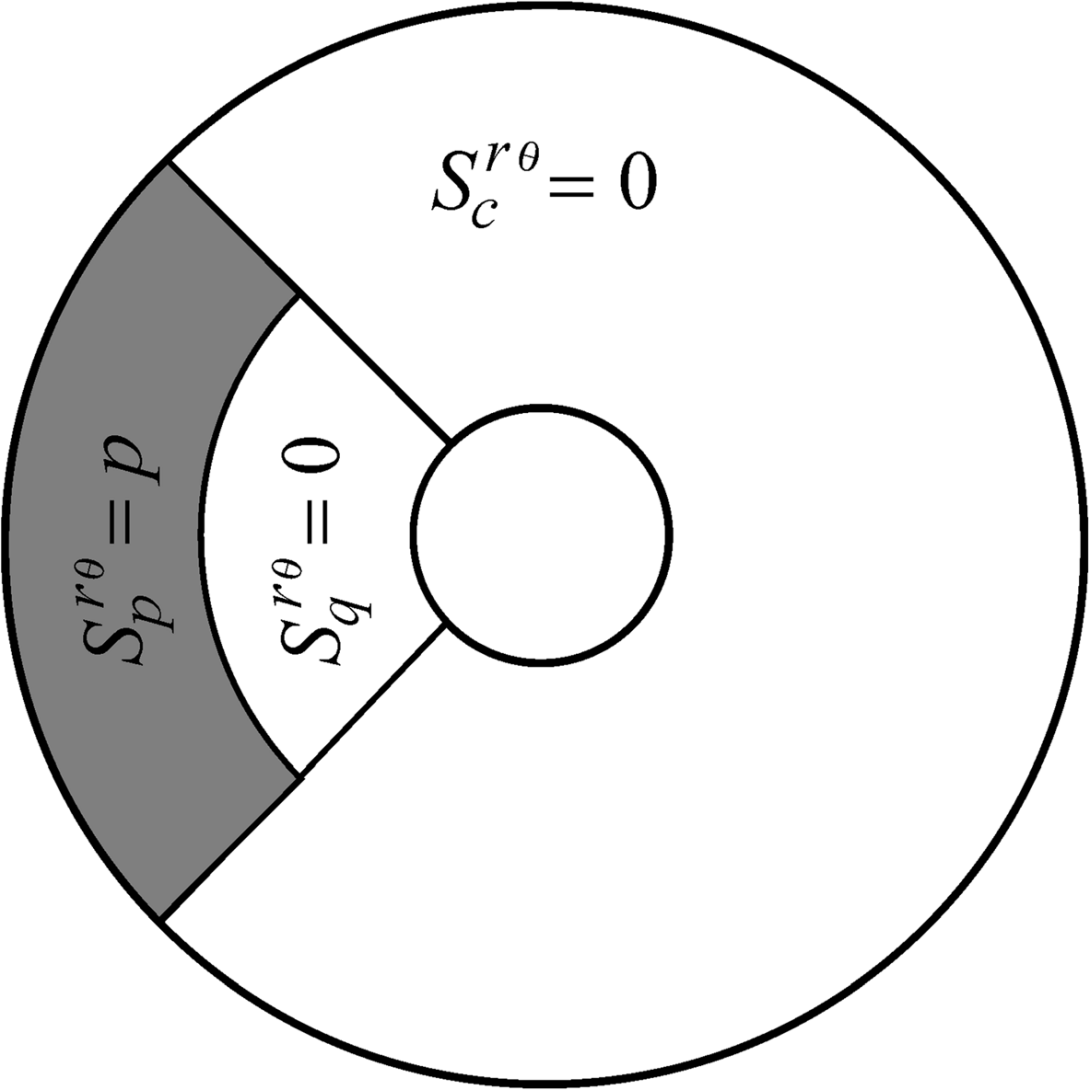
**The *p*-polarized insertion mode.** This form is symbolized here by *S*^*rθ*^(0, *p*, 0, *m*, *α*) and obtained by setting $S_{c}^{\mathrm{r\theta}}\left(m,\alpha \right)=0$, $S_{p}^{\mathrm{r\theta}}\left(m,\alpha \right)=p$, $S_{q}^{\mathrm{r\theta}}\left(m,\alpha \right)=0$, with *p* a positive constant. Under the condition *λ* = *v*_1_/2*D*_0_, which determines capping, this mode was found to produce values for *τ*_*λma*_ that are equivalent to *τ*_*du*_.

**Figure 10:**
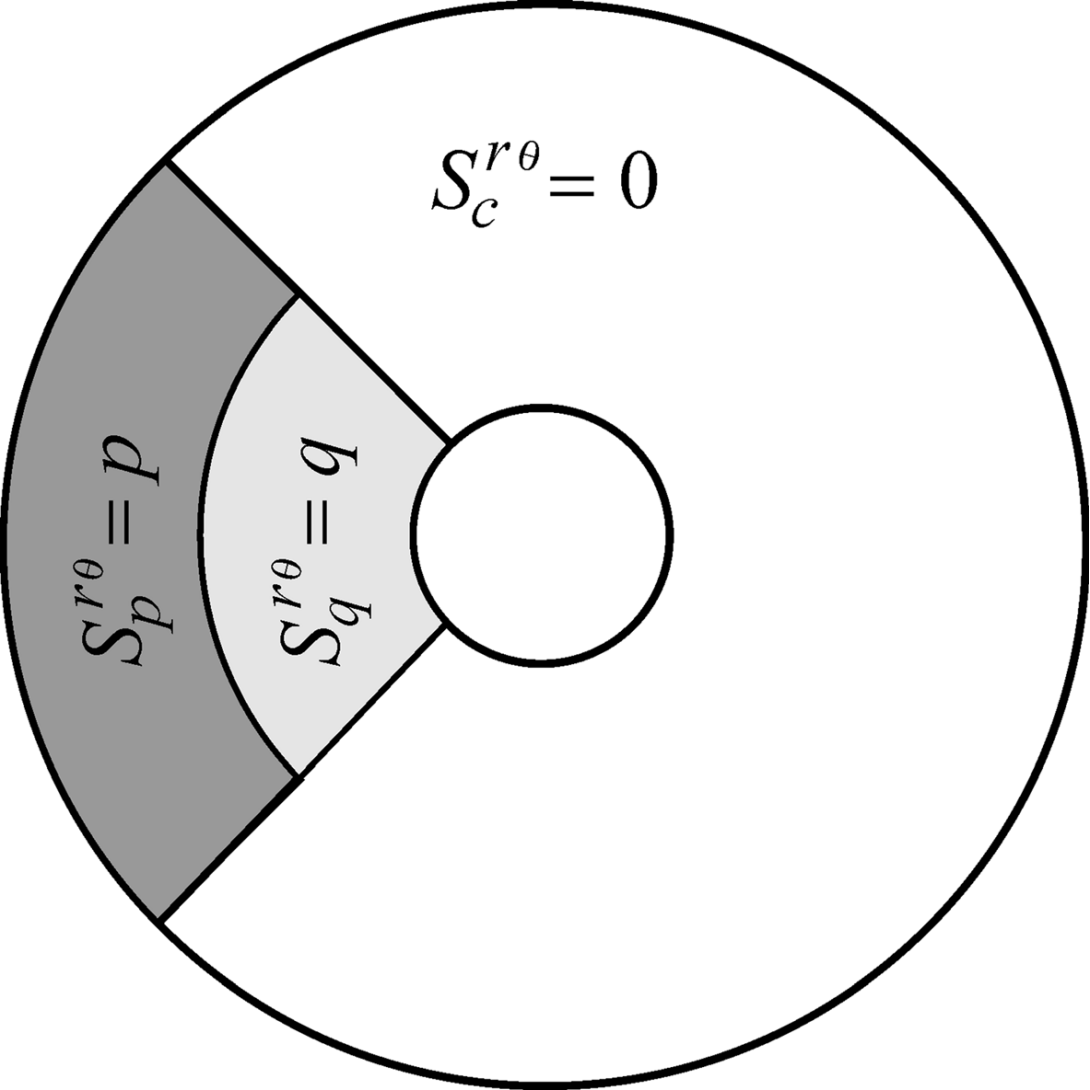
**The *pq*-polarized insertion mode.** This insertion mechanism is denoted here through *S*^*rθ*^(0, *p*, *q*, *m*, *α*). It is polarized and non-radially symmetric and linked to the conditions $S_{c}^{\mathrm{r\theta}}\left(m,\alpha \right)=0$, $S_{p}^{\mathrm{r\theta}}\left(m,\alpha \right)=p$,and $S_{q}^{\mathrm{r\theta}}\left(m,\alpha \right)=q$, with *p* and *q* as positive constant. Even for *λ* = *v*_1_/2*D*_0_, which produce capping-like effects, certain characterization of this mode can give substantial reductions in *τ*_*du*_.

**Figure 11:**
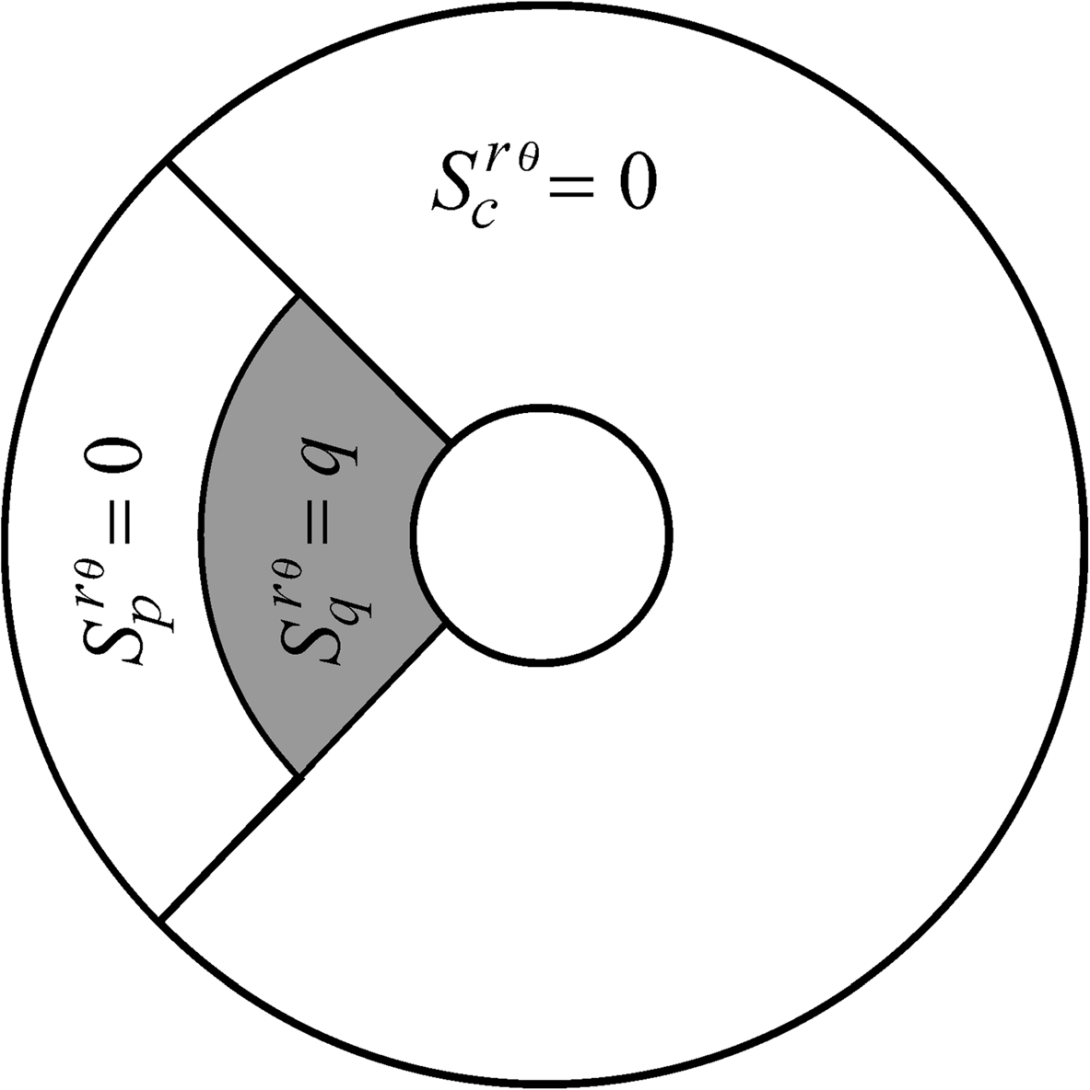
**The *q*-polarized insertion mode.** A receptor insertion paradigm denoted here by means of *S*^*rθ*^(0, 0, *q*, *m*, *α*) is linked to the case $S_{c}^{\mathrm{r\theta}}\left(m,\alpha \right)=0$, $S_{p}^{\mathrm{r\theta}}\left(m,\alpha \right)=0$, and $S_{q}^{\mathrm{r\theta}}\left(m,\alpha \right)=q$, with *q* a positive constant. This mode is non-radially symmetric and polarized. Setting = *π* , the *q*-polarized insertion mode gives the paradigm of Wofsy et al. [35] for insertion in plaques. Even when *λ* = *v*_1_/2*D*_0_ holds so that the capping phenomenon could create a graduated distribution of unbound receptors in the direction of flow streamlines, this mode seems to provide a highly efficient form of receptor insertion by making dramatic reductions on *τ*_*du*_.

We can deal with three different non-radially symmetric polarized insertion forms. One is obtained if we let $S_{c}^{\mathrm{r\theta}}\left(m,\alpha \right)=0$ and $S_{q}^{\mathrm{r\theta}}\left(m,\alpha \right)=0$, then all recycling receptors will be sorted over the region *Ω*_*p*_(*m*, *α*) and we will be dealing with a *p*-polarized receptor insertion device denoted by means of *S*^*rθ*^(0, *p*, 0, *m*, *α*) (Figure 9 and Eq. A36). Furthermore, for a *p*-polarized mode, by reducing the area of the insertion region *Ω*_*p*_(*m*, *α*), we could accommodate all recycled receptors in a favorable position relative to the cross section of the coated pit perpendicular to flow streamlines. Then, we may conjecture that this will allow the effect of convection to be maximal for receptor trapping rate enhancement. But we found that this device could at best yield a narrow variation range of *τ*_*λmα*_ relative to *τ*_*du*_ and not a relevant reduction in this value. In fact, for a *p*-polarized insertion mode with 0 < *α* ≤ *π*/6 and 1.1 ≤ *m* ≤ *b*/*a*, we found 1.01*τ*_*du*_ ≤ *τ*_*λmα*_ ≤ 1.16*τ*_*du*_. Now, if we assume that 0 < *α* < *π*, and also that $S_{c}^{\mathrm{r\theta}}\left(m,\alpha \right)=0$ hold, then receptors will be sorted over the regions *Ω*_*p*_(*m*, *α*) and *Ω*_*q*_(*m*, *α*), resulting in another form of a non-radially symmetric polarized insertion mode. This is regarded here as a *pq*-polarized receptor insertion form and denoted through *S*^*rθ*^(0, *p*, *q*, *m*, *α*) (Figure 10 and Eq. A38). In particular, for *m* = 2.2, *α* = *π*/2, *δ*_*p*_(*m*, *α*) = 0.20 and *δ*_*q*_(*m*, *π*/2) = 0.80, we calculated *τ*_*λmα*_ = 0.45*τ*_*du*_. That is, a *pq*-polarized receptor insertion form could lead to a significant reduction of *τ*_*du*_. But if we now set $S_{c}^{\mathrm{r\theta}}\left(m,\alpha \right)=0$ and $S_{p}^{\mathrm{r\theta}}\left(m,\alpha \right)=0$, then all recycling receptors will be delivered over the region *Ω*_*q*_(*m*, *α*), and we will have a third form of non-radially symmetric polarized insertion mode. This is named *q*-polarized insertion and is symbolized by means of *S*^*rθ*^(0, 0, *q*, *m*, *α*) (Figure 11 and Eq. A40). Additionally, this arrangement along with a slow convective transport (*v* = *v*_0_) and a normal diffusion process (*D* = *D*_0_)  could potentially induce a major reduction in *τ*_*du*_. In fact, if we set *m* = 2.0 and *α* = *π*/6 we obtain *τ*_*λmα*_ = 0.26*τ*_*du*_, which coincides with the plaque form insertion mode mentioned above. Hence, for the case *λ* = *v*_0_/*D*_0_ either polarized insertion over the regions *Ω*_*p*_(*m*, *α*) and *Ω*_*q*_(*m*, *α*) or over the region *Ω*_*q*_(*m*, *α*), both seem to provide an efficient mechanism for the reduction of *τ*_*du*_.

If we now assume that *λ* = *v*_1_/2*D*_0_, that is, a relatively fast convective transport (*v* = *v*_1_) and a normal diffusion process (*D* = *D*_0_), and if we again choose the *q*-polarized insertion mode *S*^*rθ*^(0, 0, 1, 2, *π*/6 ) described above, then we will get *τ*_*λmα*_ = 0.83 *τ*_*du*_. This result endorses the view that whenever convection becomes a relatively more active transport than diffusion, the process will fail to induce an effective randomization in the surface distribution of receptors [14]. The relative dominance of convection over diffusion implies that those receptors favorably distributed in an area determined by the cross section of the coated pit perpendicular to flow streamlines could be trapped, while the others would be swept away by the flow. Therefore, whenever convection is relatively fast (*v* = *v*_1_) we may assume that increasing *D*, that is, taking *D* > *D*_0_ could induce relatively greater aggregation rates in coated pits. But since we have *D*_0_ = (2.7 ± 0.09) × 10^- 9^*cm*2/*min*, then the largest feasible experimentally determined value for *D*_0_ is only *D* = 2.7910^- 9^*cm*2/*min*, and this along with the *S*^*rθ*^(0, 0, 1, 2, *π*/6 ) mode and a fast convective transport will produce *τ*_*λmα*_ = 0.75 *τ*_*du*_, which only amounts to a moderate reduction of the value *τ*_*λmα*_ = 0.83 *τ*_*du*_ calculated above. Further, if convection is relatively fast (*v* = *v*_1_) and we still have a *q*-polarized insertion mode *S*^*rθ*^(0, 0, 1, 2, *π*/6 ), then unless we have *D* = 1.74*D*_0_, the calculated value *τ*_*λmα*_ will be significantly reduced: for that arrangement we obtained *τ*_*λmα*_ = 0.24 *τ*_*du*_. Therefore, whenever convection is relatively fast (*v* = *v*_1_), even for an efficient insertion mode such as the *q*-polarized form *S*^*rθ*^(0, 0, 1, 2, *π*/6), larger values than *D* = 1.74*D*_0_ will be required for a noticeable reduction in *τ*_*du*_. Indeed, for a fast retrograde flow (*v* = *v*_1_) and a fast diffusion process (*D* = *D*_1_), we found that *S*^*rθ*^(0, 0, 1, 2, *π*/6 ) can produce a value of *τ*_*λmα*_ = 0.01*τ*_*du*_, which is a drastic reduction of *τ*_*du*_. If we assume, moreover that receptors are returned to the cell surface through a *p*-polarized insertion mode with 0 < *α* ≤ *π* and with 9.5 ≤ *m* ≤ *b*/*a*, then even if convection is extremely fast (*v* = 300*v*_1_), letting *D* = *D*_*ext*_, we get 0.19*τ*_*du*_ ≤ *τ*_*λmα*_ ≤ 0.65*τ*_*du*_. Compared with the results obtained above for the same insertion mode, this last inequality highlights the importance of diffusion in the reduction of the mean capture time of LDL receptors by coated pits. However, comparing with the expected variation range for *D*_0_, *D* values beyond *D* = 2.7910^- 8^*cm*2/*min* seem to be extremely high, and likely not achievable in the experimental system analyzed. Thus, before a faster diffusion process can be raised as an agent that can induce an effective randomization of the surface distribution of LDL receptors in the presence of a fast retrograde membrane flow, we assume instead that there must be another factor that could be adduced as a feasible device for promoting higher aggregation rates of LDL receptors in coated pits. And so, in accordance with the results presented here, we can suggest special forms of the insertion rate function *S*^*rθ*^(*c*, *p*, *q*, *m*, *α*) as mechanisms that potentially could boost the rate of removal of receptors by coated pits. Thus under the assumption of a fast convective transport, *v* = *v*_1_, and a normal diffusion process, if we allow returning receptors to be sorted by means of a *q*-polarized insertion form by setting *m* = 1.3 and *α* = *π*/2, we would get *τ*_*λmα*_ = 0.23*τ*_*du*_, implying a noticeable reduction in *τ*_*du*_. This means that whenever convection is fast and receptors diffuse normally, increasing the receptor-trapping rate could depend decidedly on polarized receptor insertion at preferential regions near coated pits.

**Figure 12:**
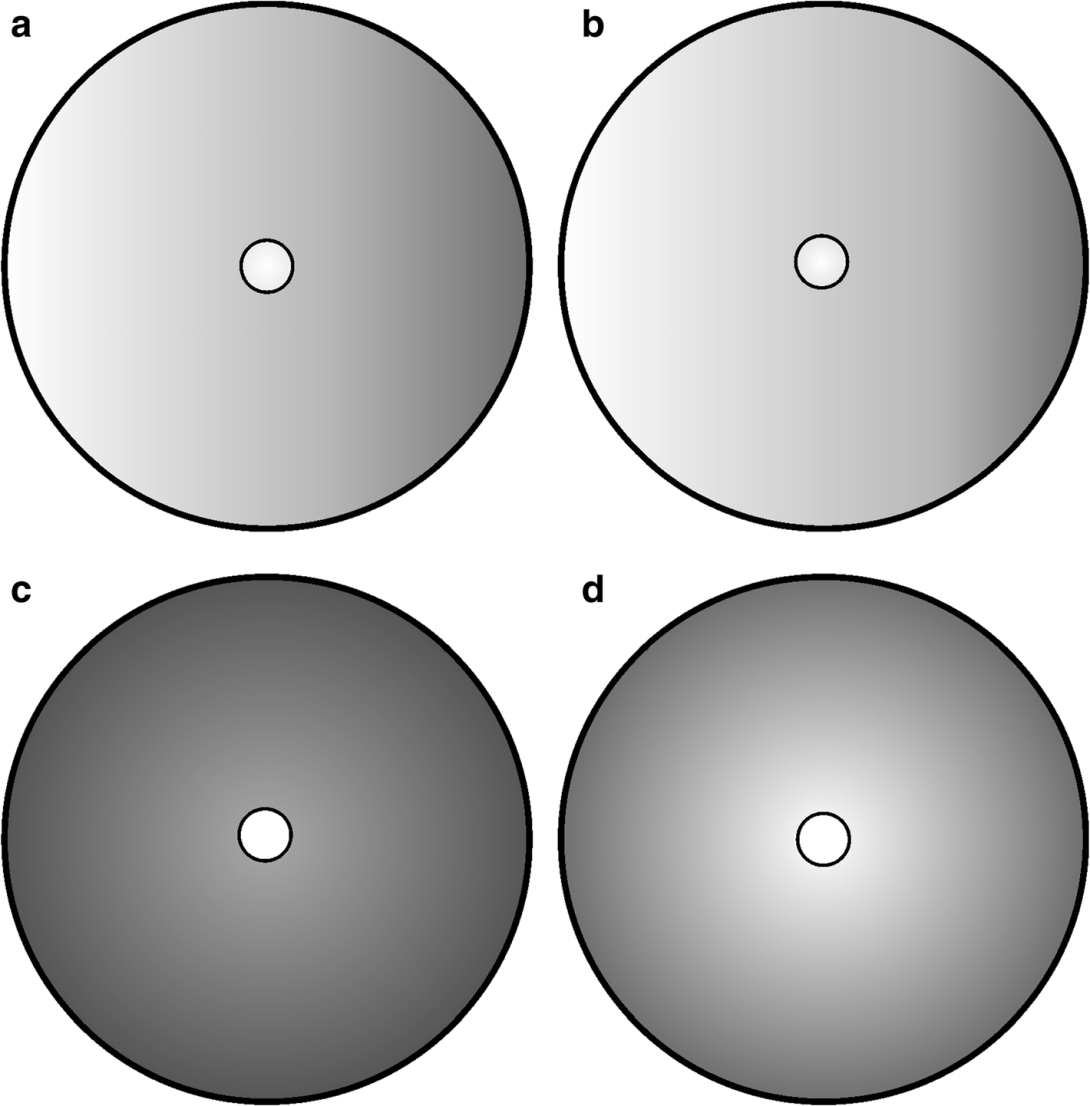
**Surface aggregation patterns of unbound LDL receptors associated with different values of the fundamental ratio λ and non-radially symmetric-polarized insertion modes. a)** the surface pattern made by *λ* = *v*_1_/2*D*_0_ and the *q*-polarized insertion mode *S*^*rθ*^(0, 0, *q*, *m*, *α*), with *m* = 2.3, *δ*_*q*_(*m*, *α*) = 1 and *α* = *π*/4; **b)** surface pattern associated with the case *λ* = *v*_1_/2*D*_1_ and the *p*-polarized insertion mode *S*^*rθ*^(0, *p*, 0, *m*, *α*) with *α* = *π*/6, *m* = 9.5, and *δ*_*p*_(*m*, *α*) = 1; **c)** the surface aggregation pattern formed by *λ* = *v*_1_/2*D*_*ext*_ and *q*-polarized insertion *S*^*rθ*^(0, *p*, *q*, *m*, *α*) with *m* = 1.2, *α* = *π*/6 and *δ*_*q*_(*m*, *α*) = 1; **d)** the surface receptor pattern for *λ* = *v*_1_/2*D*_*ext*_ and *p*-polarized insertion *S*^*rθ*^(0, *p*, *q*, *m*, *α*) with *m* = 9.5, *α* = *π*/4 and *δ*_*p*_(*m*, *α*) = 1. The patterns shown are consistent with the capping phenomena; that is, when convection is fast (*v* = *v*_1_) and diffusion is normal (*D* = *D*_0_) a graduated distribution in the direction of flow streamlines is observed, **a)** and **b)**. But in the presence of a fast convective transport, suitable values of the diffusion coefficient (e. g. *D* = *D*_*ext*_) can reverse the capping effect and induce an effective randomization of the distribution of unbound LDL receptors, **c)** and **d)**. In any event, in the presence of a retrograde membrane flow, no receptor surface clusters are formed.

For the insertion-transport mechanism for LDL receptors under consideration, we also address here the theoretical exploration of their consequent display on the cell surface. A capping-like surface display should manifest as a graduated concentration of receptors in the direction of flow streamlines. Figure 12 displays the surface aggregation patterns of receptors that result when a polarized reinsertion mode is considered. The combination of polarized insertion, fast convective transport of rate *v* = *v*_1_ and a relatively slow diffusion process such as one associated to *D*_0_ can be observed to produce a marked gradient in the distribution of unbound receptors (Figure 12a and b). This is consistent with the conceptual model for the capping phenomenon presented by Bretscher [14]. Further, and in concurrence with that author, the present model explains this gradient as being induced by a relative dominance of convection over diffusion; that is, an arrangement which precludes an effective randomization of the surface-receptor distribution. Thus, in the presence of a retrograde membrane flow with typical strength *v* = *v*_1_, even single LDL particles having a diffusion coefficient value *D* = *D*_0_ —which is considered normal—would produce a capping-like cell surface distribution; thus precluding both a uniform distribution and the display of clusters of unbound receptors on the surface. Even in the presence of a fast convective transport, a suitably fast diffusion process can reverse the capping effects (Figures 12c and d).

Our results are mainly consistent with the capping phenomenon, and suggest that this is indeed concomitant with the idea of a retrograde membrane flow. Capping is described as the rearward transport across the leading lamella of various materials used to mark the cell surface [46,14]. Bretscher [14] asserts that during capping, those receptors that are excluded by coated pits, and therefore do not recycle, may be continuously swept by a retrograde membrane flow. This author also states that whether such a non-circulating protein does get swept backwards or not depends on how fast it can diffuse by Brownian motion and how fast the flow is; and moreover that for individual proteins to overcome the sweeping effect of the flow, they must have diffusion coefficients (*D*) of about *D*_*ext*_[14]. This assertion was corroborated by our results, since our model predicts that for *D* values of the order of *D*_*ext*_ the capping effect can be reversed. Nevertheless, a value of *D*_*ext*_ lies far beyond the maximum experimentally feasible value for *D*_0_. And even though Bretscher [14] claims that FBR often underestimates the true value of the diffusion coefficients of pertinent surface receptors, a value *D*_*ext*_  for *D* in the LDL experimental system seems unfeasible. Our results suggest that this is where the relative importance of non-uniform insertion becomes evident as a factor for boosting LDL receptor trapping. Indeed, even when convection is fast and receptors diffuse normally, our findings indicate that special forms of polarized receptor insertion can be arranged so as to produce a substantial reduction in *τ*_*du*_. This was more obvious in the case of a *q*-polarized mode, which could be raised as a feasible preferential-insertion mechanism for enhancing the LDL receptor trapping rate.

The problem of the surface distribution of unbound receptors is an important question pertaining to the LDL experimental system. For example, Wofsy et al*.*[40] argued that in the Robenek and Hesz [39] experiments, the LDL-gold particles were highly multivalent and thus may have been bound more efficiently to aggregate than single receptors, suggesting that the aggregation of newly inserted LDL receptors in regions around coated pits was still unproven. But beyond the caveats of Wofsy et al. [40] the results of Gross and Webb [47] were considered to provide a quantitative basis in support of a surface distribution of both LDL particles and their receptors in different-sized clusters. And again, the existence of these clusters was questioned by Sanan et al. [48] who reported experimental results that detected a dispersed or scattered population of LDL receptors, in addition to and clearly distinct from, clusters that formed soon after their insertion during recycling. In view of these disparate reports, Robenek et al. [49] performed new experiments on fibroblast and hepatocyte plasma membranes which confirmed their original claim that the initial insertion and display of LDL receptors in fibroblasts occurs predominantly as plaques. But experiments using fluorescently labeled LDL and influenza virus particles bound to the surface of human fibroblasts imaged with a cooled-scan CCD camera attached to a fluorescence microscope were found to yield a reasonably accurate measure of the proportion of single particles, but large errors were encountered in the proportion of large cluster sizes [50]. The high proportion of single particles found in these data provided evidence against the clusters claimed by Robenek et al. [49] and support the random insertion model. It is still a matter of disagreement among different research groups whether newly inserted LDL receptors are dispersed in the plasma membrane of cultured fibroblasts prior to entrapment in coated pits or whether they remain after reinsertion as groups of clusters. Our findings reveal that in the presence of a fast retrograde membrane flow, the expected surface display of unbound receptors must be consistent with the capping phenomenon; thus, the formation of steady-state surface clumps of LDL receptors is unachievable in the current setting (Figure 12).

Traditionally the existence of a retrograde flow has been a source of controversy among researchers, some of whom disclaim this paradigm (e.g. [51-53]) while others convincingly address it (e.g. [14,54,55]). In particular, our results provide a theoretical support for the claim of Bretscher [14] which suggests that, if a retrograde flow does exist, the diffusion-convection ratio could be a fundamental determinant of the surface display of LDL receptors that are not bound to coated pits. And even though biochemical or biomechanical factors have been invoked in order to put forward other paradigms for the capping phenomenon (e.g. [49,56-58]), our findings support the views of Bretscher [59] and Ishihara et al. [60] on the adequacy of the retrograde-flow model. Hence, the involvement of diffusion, polarized reinsertion and a retrograde membrane flow in the formalization of the receptor-mediated endocytic cycle may provide a trustworthy paradigm for the interpretation of observations. Moreover, the fact that some characterizations of the retrograde flow-diffusion ratio could induce slower receptor trapping rates cannot provide a rebuttal for the retrograde-flow model. Indeed, Echavarria-Heras et al. [41] suggested that receptors could be projected to the plasma membrane in a preferential mode relative to the flow streamlines. We have studied here the consequences of such an arrangement and our results show that certainly, even in the presence of a fast retrograde flow, we could consider a *q*-polarized form as a mechanism for the enhancement of LDL receptor trapping rates.

## Conclusions

Our results seem to indicate that even in the presence of a fast retrograde membrane flow, which could influence capping-like effects, diffusing LDL receptors that are sorted through polarized insertion in the form conceptualized here, could spur faster aggregation rates in coated pits than those associated with uniform insertion over the entire cell membrane. Moreover a *q*-polarized form could provide a paradigm for a highly efficient form of preferential insertion. The effectiveness of this mode is mainly explained by its placement relative to the flow streamlines. Furthermore, if the cross sections of the linked insertion region *Ω*_*q*_(*m*, *α*) and those associated with a coated pit are comparable, then even though a relative dominance of convection over diffusion might occur, the privileged position of the insertion region *Ω*_*q*_(*m*, *α*) would induce a fast receptor-trapping rate. But our results also hint that in spite of the efficiency of this device, the influence of a retrograde flow would cancel out the display of the corresponding surface clusters. Moreover, if new experiments do reveal that the surface clusters exist, then it could be necessary to envision a mechanism that explains their formation in the presence of a retrograde membrane flow. Meanwhile, for the case *v* = *v*_1_ and *D* = *D*_0_, a *q*-polarized insertion mode with *m* = 1.20 and *α* = *π*/16 provides a convenient paradigm for explaining the coexistence of a retrograde membrane flow and receptor aggregations that might be experimentally misinterpreted as surface clusters. Indeed, considering the experimental error associated with the measurement of the coated pit radius, a region of insertion of the form *Ω*_*q*_  with *m* = 1.20 could actually be a region placed over the boundary of a coated pit. And the surface clusters observed might be formed when recycling receptors are returned near the periphery of newly-formed coated pits so as to enhance the removal of the LDL ligand, but the existence of that insertion paradigm would require experimental substantiation.

## Appendix A

In accordance with Eq. (17), the function *S*^*rθ*^(*c*, *p*, *q*, *m*, *α*) inserts $S_{*}^{\mathrm{r\theta}}\left(m,\alpha \right)$ particles per unit area per unit time at (*r*, *θ*)  in Ω_*_. Suppose that the number of particles that $S_{*}^{\mathrm{r\theta}}\left(m,\alpha \right)$ inserts in Ω_*_ is I_*_(*m*, *α*). Then, we have

(A1) $$\int_{a}^{b}\int_{-\pi +\alpha }^{\pi -\alpha }S_{c}^{\mathrm{r\theta}}\left(m,\alpha \right)\;\mathrm{rdrd\theta}=I_{c\;}\left(m,\alpha \right),$$

(A2) $$\int_{\mathrm{ma}}^{b}\int_{\pi -\alpha }^{\pi +\alpha }S_{p}^{\mathrm{r\theta}}\left(m,\alpha \right)\;\mathrm{rdrd\theta}=I_{p}\left(m,\alpha \right),$$

(A3) $$\int_{a}^{\mathrm{ma}}\int_{\pi -\alpha }^{\pi +\alpha }S_{q}^{\mathrm{r\theta}}\left(m,\alpha \right)\;\mathrm{rdrd\theta}=I_{q}\left(m,\alpha \right).$$

Let's denote by means of *I*_*Ω*_ the total number of particles inserted by *S*^*rθ*^(*c*, *p*, *q*, *m*, *α*) in *Ω*. Assume also, that a proportion *δ*_*p*_(*m*, *α*) of the total number of particles inserted in *Ω* is sorted by *S*^*rθ*^(*c*, *p*, *q*, *m*, *α*) in *Ω*_*p*_(*m*, *α*), that a proportion *δ*_*q*_(*m*, *α*) of $I_{Ω}$ is placed into the region *Ω*_*q*_(*m*, *α*), and that a proportion *δ*_*c*_(*m*, *α*) of *I*_*Ω*_ is delivered in *Ω*_*q*_(*m*, *α*). Then, we must have

(A4) $$I_{c}\;\left(m,\alpha \right)=\delta_{c}\left(m,\alpha \right)I_{\Omega },$$

(A5) $$I_{p}\left(m,\alpha \right)=\delta_{p}\left(m,\alpha \right)I_{\Omega },$$

(A6) $$I_{q}\left(m,\alpha \right)=\delta_{q}\left(m,\alpha \right)I_{\Omega },$$

and the balance equation

(A7) $$\delta_{p}\left(m,\alpha \right)+\delta_{q}\left(m,\alpha \right)+\delta_{c}\left(m,\alpha \right)=1.$$

From equations (A1) through (A3) and the mean value theorem there are numbers

(A8) $$\left(r_{c},\theta_{c}\right)\in \Omega_{c}\left(m,\alpha \right),$$

(A9) $$\left(r_{p},\theta_{p}\right)\in \Omega_{p}\left(m,\alpha \right),$$

(A10) $$\left(r_{q},\theta_{q}\right)\in \Omega_{q}\left(m,\alpha \right),$$

such that

(A11) $$\delta_{p}\left(m,\alpha \right)=\frac{\alpha S_{p}^{r_{p}\theta_{p}}\left(m,\alpha ,\right)\left(b^{2}-a^{2}m^{2}\right)}{I_{\Omega }},$$

(A12) $$\delta_{q}\left(m,\alpha \right)=\frac{\alpha S_{q}^{r_{q}\theta_{q}}\left(m,\alpha ,\right)\left(a^{2}m^{2}-a^{2}\right)}{I_{\Omega }},$$

(A13) $$\delta_{c}\left(m,\alpha \right)=\frac{\alpha S_{c}^{r_{c}\theta_{c}}\left(m,\alpha ,\right)\left(\;\pi -\alpha \right)\left(b^{2}-a^{2}\right)}{I_{\Omega }}.$$

Therefore, we have to introduce the consistency conditions

(A14) $$\delta_{p}\left(b/a,\alpha \right)=0,$$

(A15) $$\delta_{p}\left(m,0\right)=0,$$

(A16) $$\delta_{p}\left(1,\pi \right)=1,$$

(A17) $$\delta_{q}\left(1,\alpha \right)=0,$$

(A18) $$\delta_{q}\left(b/a,\pi \right)=1,$$

(A19) $$\delta_{q}\left(m,0\right)=0,$$

(A20) $$\delta_{c}\left(m,\pi \right)=0,$$

(A21) $$\delta_{c}\left(m,0\right)=1.$$

A uniform insertion mode is obtained by setting $S_{c}^{\mathrm{r\theta}}\left(b/a,0\right)=c$, where *c* is a positive constant. This is symbolically represented by means of *S*^*rθ*^(*c*, 0, 0, *b*/*a*, 0) and given by

(A22) $$\;S^{\mathrm{r\theta}}\left(c,0,0,b/a,0\right)=\left\{\begin{array}{cc} c & \left(r,\theta \right)\in \Omega_{c}\left(b/a,0\right)\\ 0 & \left(r,\theta \right)\in \Omega_{p}\left(b/a,0\right)\\ 0 & \left(r,\theta \right)\in \Omega_{q}\left(b/a,0\right) \end{array}\right),$$

where

(A23) $$c=\frac{I_{\Omega }}{\pi \left(b^{2}-a^{2}\right)}.$$

Also, uniform insertion is associated in the case $S_{c}^{\mathrm{r\theta}}\left(m,\pi \right)=0$, $S_{p}^{\mathrm{r\theta}}\left(m,\pi \right)=S_{q}^{\mathrm{r\theta}}\left(m,\pi \right)=c.$ This mode is known as *pq*-uniform insertion mode and denoted by means of *S*^*rθ*^(0, *p*, *q*, *m*, *π*),

(A24) $$\;S^{\mathrm{r\theta}}\left(0,c,c,m,\pi \right)=\left\{\begin{array}{cc} 0 & \;\left(r,\theta \right)\in \Omega_{c}\left(m,\pi \right)\\ c & \left(r,\theta \right)\in \Omega_{p}\left(m,\pi \right)\\ c & \left(r,\theta \right)\in \Omega_{q}\left(m,\pi \right) \end{array}\right).$$

Again, from equations (A11) through (A13),

(A25) $$c=\frac{I_{\Omega }}{\alpha \left(b^{2}-a^{2}\right)}.$$

Moreover, the insertion rate function

(A26) $$\;S^{\mathrm{r\theta}}\left(c,c,c,m,\alpha \right)=\left\{\begin{array}{cc} c & \;\left(r,\theta \right)\in \Omega_{c}\left(m,\alpha \right)\\ c & \left(r,\theta \right)\in \Omega_{p}\left(m,\alpha \right)\\ c & \left(r,\theta \right)\in \Omega_{q}\left(m,\alpha \right) \end{array}\right),$$

which is obtained for the case $S_{c}^{\mathrm{r\theta}}\left(m,\alpha \right)=S_{p}^{\mathrm{r\theta}}\left(m,\alpha \right)=S_{q}^{\mathrm{r\theta}}\left(m,\alpha \right)=c$, also inserts particles uniformly all over *Ω*. This is known as a *cpq*-uniform insertion mode. From equations (A11) through (A13) we have

(A27) $$c=\frac{I_{\Omega }}{\alpha \left(b^{2}-a^{2}m^{2}\right)+\left(\pi -\alpha \right)\left(b^{2}-a^{2}\right)+\alpha \left(a^{2}m^{2}-a^{2}\right)}.$$

The form of *S*^*rθ*^(*c*, *p*, *q*, *m*, *α*) obtained by setting $S_{c}^{\mathrm{r\theta}}\left(m,\alpha \right)=c$, $S_{p}^{\mathrm{r\theta}}\left(m,\alpha \right)=p$ and $S_{q}^{\mathrm{r\theta}}\left(m,\alpha \right)=q$, where *c, p* and *q* are positive constants will be known as a *cpq*-locally uniform insertion mode. That is, from equation (17) we will have

(A28) $$\;S^{\mathrm{r\theta}}\left(c,p,q,m,\alpha \right)=\left\{\begin{array}{cc} c & \left(r,\theta \right)\in \Omega_{c}\left(m,\alpha \right)\\ p & \left(r,\theta \right)\in \Omega_{p}\left(m,\alpha \right)\\ q & \left(r,\theta \right)\in \Omega_{q}\left(m,\alpha \right) \end{array}\right)$$

with

(A29) $$\frac{\mathrm{\alpha p}\left(b^{2}-a^{2}m^{2}\right)}{I_{\Omega }}+\frac{\mathrm{\alpha q}\left(a^{2}m^{2}-a^{2}\right)}{I_{\Omega }}+\frac{\mathrm{\alpha c}\left(\;\pi -\alpha \right)\left(b^{2}-a^{2}\right)}{I_{\Omega }}=1.$$

A second form of locally uniform insertion denoted by means of *S*^*rθ*^(0, *p*, *q*, *m*, *π*) and known as a *pq*-locally uniform insertion mode is obtained for the case $S_{c}^{\mathrm{r\theta}}\left(m,\pi \right)=0$, $S_{p}^{\mathrm{r\theta}}\left(m,\pi \right)=p$ and $S_{q}^{\mathrm{r\theta}}\left(m,\pi \right)=q$, that is,

(A30) $$\;S^{\mathrm{r\theta}}\left(0,p,q,m,\pi \right)=\left\{\begin{array}{cc} 0 & \left(r,\theta \right)\in \Omega_{c}\left(m,\pi \right)\\ p & \left(r,\theta \right)\in \Omega_{p}\left(m,\pi \right)\\ q & \left(r,\theta \right)\in \Omega_{q}\left(m,\pi \right) \end{array}\right)$$

where, from equations (A11) through (A13) *p* and *q* satisfy

(A31) $$\frac{\mathrm{\alpha p}\left(b^{2}-a^{2}m^{2}\right)}{I_{\Omega }}+\frac{\mathrm{\alpha q}\left(a^{2}m^{2}-a^{2}\right)}{I_{\Omega }}=1.$$

The case in which receptors are inserted into two disjoint regions of abruptly contrasting rates is defined as a polarized insertion mode. It will be typified here as a mode that sorts the total number of recycled receptors over either the region *Ω*_*p*_(*m*, *α*), or *Ω*_*q*_(*m*, *α*), or *Ω*_*p*_(*m*, *α*) ∪ *Ω*_*q*_(*m*, *α*). Moreover, a polarized insertion mode can be obtained in five different forms, two of these forms being radially symmetric.

The insertion rate function known as a *q*-plaque form is denoted by means of *S*^*rθ*^(0, 0, *q*, *m*, *π*) and obtained for $S_{c}^{\mathrm{r\theta}}\left(m,\pi \right)=0$, $S_{p}^{\mathrm{r\theta}}\left(m,\pi \right)=0$, and $S_{q}^{\mathrm{r\theta}}\left(m,\pi \right)=q$, being *q* a positive constant, that is,

(A32) $$\;S^{\mathrm{r\theta}}\left(0,0,q,m,\pi \right)=\left\{\begin{array}{cc} 0 & \left(r,\theta \right)\in \Omega_{c}\left(m,\alpha \right)\\ 0 & \left(r,\theta \right)\in \Omega_{p}\left(m,\alpha \right)\\ q & \left(r,\theta \right)\in \Omega_{q}\left(m,\alpha \right) \end{array}\right)$$

is radially symmetric and polarized, and from equations (A11) through (A13) we have

(A33) $$q=\frac{I_{\Omega }}{\pi \left(a^{2}m^{2}-a^{2}\right)}.$$

The function *S*^*rθ*^(0, 0, *q*, *m*, *π*) moreover, coincides with the plaque form insertion mode proposed by Wofsy et al. [40]. And, by setting *S*^*rθ*^(0, 0, *q*, *m*, *π*) = *Sr*^- *β*^ one obtains from equation (17) the decreasing and radially symmetric insertion-rate function considered by Echavarria-Heras et al. [41].

Further, what we call here a *p*-peripheral insertion mode was proposed by Bretscher [38]. It can be also considered as a radially symmetric polarized insertion mode. It is denoted through *S*^*rθ*^(0, *p*, 0, *m*, *π*) and is obtained by setting $S_{c}^{\mathrm{r\theta}}\left(m,\pi \right)=0$, $S_{p}^{\mathrm{r\theta}}\left(m,\pi \right)=p$ and $S_{q}^{\mathrm{r\theta}}\left(m,\pi \right)=0$, with *p* a positive constant, that is

(A34) $$\;S^{\mathrm{r\theta}}\left(0,p,0,m,\pi \right)=\left\{\begin{array}{cc} 0 & \left(r,\theta \right)\in \Omega_{c}\left(m,\pi \right)\\ p & \left(r,\theta \right)\in \Omega_{p}\left(m,\pi \right)\\ 0 & \left(r,\theta \right)\in \Omega_{q}\left(m,\pi \right) \end{array}\right),$$

and from equations (A11) through (A13) we have

(A35) $$p=\frac{I_{\Omega }}{\pi \left(b^{2}-a^{2}m^{2}\right)}.$$

A first form of a non-radially symmetric polarized insertion mode is known here as *p*-polarized. It is denoted by means of *S*^*rθ*^(0, *p*, 0, *m*, *α*)  and arises whenever $S_{c}^{\mathrm{r\theta}}\left(m,\alpha \right)=0$, $S_{p}^{\mathrm{r\theta}}\left(m,\alpha \right)=p$ and $S_{q}^{\mathrm{r\theta}}\left(m,\alpha \right)=0$, where *p* is a positive constant, that is,

(A36) $$\;S^{\mathrm{r\theta}}\left(0,p,0,m,\alpha \right)=\left\{\begin{array}{cc} 0 & \left(r,\theta \right)\in \Omega_{c}\left(m,\alpha \right)\\ p & \left(r,\theta \right)\in \Omega_{p}\left(m,\alpha \right)\\ 0 & \left(r,\theta \right)\in \Omega_{q}\left(m,\alpha \right) \end{array}\right)$$

where from equations (A11) through (A13) we have

(A37) $$p=\frac{I_{\Omega }}{\alpha \left(b^{2}-a^{2}m^{2}\right)}.$$

A second form of a non-radially symmetric polarized receptor insertion mode is known here as *pq*-polarized. It is denoted by means of *S*^*rθ*^(0, *p*, *q*, *m*, *α*), and is linked to the case $S_{c}^{\mathrm{r\theta}}\left(m,\alpha \right)=0$, $S_{p}^{\mathrm{r\theta}}\left(m,\alpha \right)=p$, and $S_{q}^{\mathrm{r\theta}}\left(m,\alpha \right)=q$, with *p* and *q* positive constants, that is,

(A38) $$\;S^{\mathrm{r\theta}}\left(0,p,q,m,\alpha \right)=\left\{\begin{array}{cc} 0 & \left(r,\theta \right)\in \Omega_{c}\left(m,\alpha \right)\\ p & \left(r,\theta \right)\in \Omega_{p}\left(m,\alpha \right)\\ q & \left(r,\theta \right)\in \Omega_{q}\left(m,\alpha \right) \end{array}\right)$$

and from equations (A11) through (A13) we have

(A39) $$\frac{I_{\Omega }p\left(m,\alpha \right)}{\alpha \left(b^{2}-a^{2}m^{2}\right)}+\frac{I_{\Omega }q\left(m,\alpha \right)}{\alpha \left(a^{2}m^{2}-a^{2}\right)}=1.$$

A third non-radially symmetric and polarized form called *q*-polarized is symbolized by means of *S*^*rθ*^(0, 0, *q*, *m*, *α*). It is linked to the case $S_{c}^{\mathrm{r\theta}}\left(m,\alpha \right)=0$, $S_{p}^{\mathrm{r\theta}}\left(m,\alpha \right)=0$ and $S_{q}^{\mathrm{r\theta}}\left(m,\alpha \right)=q$, where *q* is a positive constant, that is,

(A40) $$\;S^{\mathrm{r\theta}}\left(0,0,q,m,\alpha \right)=\left\{\begin{array}{cc} 0 & \left(r,\theta \right)\in \Omega_{c}\left(m,\alpha \right)\\ 0 & \left(r,\theta \right)\in \Omega_{p}\left(m,\alpha \right)\\ q & \left(r,\theta \right)\in \Omega_{q}\left(m,\alpha \right) \end{array}\right)$$

where from equations (A11) through (A13) we have

(A41) $$q=\frac{I_{\Omega }}{\alpha \left(a^{2}m^{2}-a^{2}\right)}.$$

## Appendix B

In this section we outline the adaptation of the model for receptor mediated endocytosis presented in Echavarria-Heras et al. [41] for the case of a partitioned receptor insertion mode. For detailed mathematical proofs the reader is referred to Echavarria-Heras and Leal-Ramirez [61]. In the present settings the reference region $Ω=\left\{\left(x,y\right)|a\leq \sqrt{x^{2}+y^{2}}\leq b\right\}$ coincides with the Berg-Purcell [23] geometry. Equation (5) scales the outer radius *b*. A retrograde membrane flow will be represented by means of the vector field $\vec{V}\left(x,y,t\right)=v\;\vec{i}$ with $\vec{i}$ being the unitary vector pointing in the direction of the positive *x*-axis and *v*, the strength of the flow in units of *cm*/*s*. Since we have assumed here a convective diffusion transport and also a partitioned receptor-insertion mode (cf. Eq. (17)) we must extend the model of equations (20) through (22) in order to include these mechanisms. The symbol *D* will stand for the 2-dimensional diffusion coefficient, which has units of *cm*^2^/*s* and will be assumed to be constant. The ratio

(B1) $$\lambda =\frac{v}{2D},$$

having units *cm*^- 1^ is known as the fundamental ratio of the transport process because it combines the parameters *v* and *D*, which represent the factors that control receptor movement on the cell surface. Also, *S*(*m*, *α*, *x*, *y*)  will denote the rate of particle insertion, with units: *particles*/*cm*^2^ - *s*. At steady state, the corresponding concentration of particles *C*(*λ*, *x*, *y*) satisfies the partial differential equation in the annulus *Ω*[62],

(B2) $$-\mathrm{div}\left(D\nabla C\right)+\mathrm{div}\left(C\vec{V}\right)=S\left(m,\alpha ,x,y\right)$$

Equivalently,

(B3) $$D\nabla^{2}C-v\frac{\partial C}{\partial x}+S\left(m,\alpha ,x,y\right)=0.$$

Due to the geometry determined by *Ω*, it is convenient to switch to polar coordinates. Then, if we denote by means of $\vec{n}$ the normal unit vector pointing out radially towards (*r*, *θ*) for 0 ≤ *θ* ≤ 2*π*, and if $\vec{J}$ stands for the flux vector, the projection of the flux vector over $\vec{n}$ is given by

(B4) $$\vec{J}∙\vec{n}=D\frac{\partial C}{\partial x}+\mathrm{vC}\cos\theta .$$

We will consider the partitioned insertion mode of equation (17), that is

(B5) $$S\left(m,\alpha ,r,\theta \right)=\left\{\begin{array}{cc} S_{c}^{\mathrm{r\theta}}\left(m,\alpha \right) & \left(r,\theta \right)\in \Omega_{c}\left(m,\alpha \right)\\ S_{p}^{\mathrm{r\theta}}\left(m,\alpha \right) & \left(r,\theta \right)\in \Omega_{p}\left(m,\alpha \right)\\ S_{q}^{\mathrm{r\theta}}\left(m,\alpha \right) & \left(r,\theta \right)\in \Omega_{q}\left(m,\alpha \right) \end{array}\right).$$

Because of the nature of the convective vector field *C*_*λmα*_(*r*, *θ*) is not expected to be radially symmetric if the strength of the membrane flow is different from zero. However, *C*_*λmα*_(*r*, *θ*)  will be symmetric about the x-axis. This property is formally expressed by

(B6) $$C_{\mathrm{\lambda m\alpha}}\left(r,\theta \right)=C_{\mathrm{\lambda m\alpha}}\left(r,-\theta \right).$$

The boundary at *r* = *a* remains as absorbing, i.e.

(B7) $$C_{\mathrm{\lambda m\alpha}}\left(r,\theta \right)|{}_{r=a}=0.$$

On the other hand, the reflecting boundary condition given by equation (8) must be modified in order to account for the influence of the retrograde flow. We will consider that this boundary condition can be replaced by a flux-vanishing boundary condition at *r* = *b*. Hence the boundary condition at *r* = *b* will be formally represented by means of the equation

(B8) $$\int_{\partial Ωb}\vec{J}∙\vec{n}dΩ=0.$$

Where ∂*Ωb* denotes the outer boundary of the annulus *Ω*, since we have assumed a steady-state for the receptors. This requirement for a vanishing flux of particles across the boundary at *r* = *b* can be considered a model for a dynamics in which those receptors crossing the boundary at *r* = *b* are transported into a contiguous influence area determined by the direction of the flow streamlines.

The further formal treatment can be greatly simplified by introducing a function *G*_*λmα*_(*r*, *θ*) defined through,

(B9) $$G_{\mathrm{\lambda m\alpha}}\left(r,\theta \right)=C_{\mathrm{\lambda m\alpha}}\left(r,\theta \right)e^{-\mathrm{\lambda r}\cos\theta }.$$

The substitution of Eq. (B9) into Eq. (B3) and the fulfilment of the symmetry and boundary conditions for *C*_*λmα*_(*r*, *θ*) show that, *G*_*λmα*_(*r*, *θ*) must satisfy

(B10) $$\nabla^{2}G_{\mathrm{\lambda m\alpha}}\left(r,\theta \right)-\lambda^{2}G_{\mathrm{\lambda m\alpha}}\left(r,\theta \right)=-\frac{S^{\mathrm{r\theta}}\left(c,p,q,m,\alpha \right)\;}{D}e^{-\mathrm{\lambda b}\cos\theta },$$

(B11) $$G_{\mathrm{\lambda m\alpha}}\left(a,\theta \right)=0,$$

(B12) $$G_{\mathrm{\lambda m\alpha}}\left(r,\theta \right)=G_{\mathrm{\lambda m\alpha}}\left(r,-\theta \right),$$

(B13) $$G_{\mathrm{\lambda m\alpha}}\left(r,\theta \right)\;\mathrm{periodic}\;\mathrm{in}\;\theta ,$$

(B14) $$\int_{-\pi }^{\pi }\left(\frac{\partial G_{\mathrm{\lambda m\alpha}}\left(r,\theta \right)}{\partial r}|{}_{r=b}-\lambda \cos\theta G_{\mathrm{\lambda m\alpha}}\left(b,\theta \right)\right)e^{-\mathrm{\lambda b}\cos\theta }\mathrm{d\theta}=0.$$

Finally, for consistency a limiting radial symmetry condition must be added when the strength of the flow approaches zero. That is, we will require that for fixed *D* > 0, *G*_*λmα*_(*r*, *θ*) satisfies

(B15) $$\lim_{\lambda \to 0}G_{\mathrm{\lambda m\alpha}}\left(r,\theta \right)=C_{\mathrm{ds}}\left(r\right)$$

Where *C*_*ds*_(*r*) is the solution of equation (6) subject to boundary conditions (7) and (8).

Using the boundary conditions (B11) trough (B14) it is possible to obtain, using separation of variables, a solution *G*_*λmα*_(*r*, *θ*) for equation (B10). Furthermore *G*_*λmα*_(*r*, *θ*) can be written in the form,

(B16) $$G_{\mathrm{\lambda m\alpha}}\left(r,\theta \right)=\sum_{k=0}^{\infty }g_{\mathrm{\lambda m\alpha}}^{k}\left(r\right)\cos\mathrm{k\theta},$$

where for *k* = 0, 1 …, *a* ≤ *m* ≤ *b*/*a*, 0 ≤ *α* ≤ *π* and *λ* ∈ *R*, the function $g_{\mathrm{\lambda m\alpha}}^{k}\left(r\right)$ satisfies the non-homogeneous problem

(B17) $$L_{\lambda }^{k}\left(u\right)+\lambda^{2}u=\frac{\sigma_{k}}{D}\int_{-\pi }^{\pi }S^{\mathrm{r\theta}}\left(c,p,q,m,\alpha \right)e^{-\mathrm{\lambda r}\cos\theta }\cos\mathrm{k\theta d\theta},$$

with

(B18) $$\sigma_{k}=\left\{\begin{array}{cc} \frac{1}{2\pi } & k=0\\ \frac{1}{\pi } & k=1,2,\ldots , \end{array}\right),$$

and

(B19) $$L_{\lambda }^{k}\left(u\right)=\frac{1}{r}\left(-{\left(u^{'}\right)}^{'}+\frac{k^{2}}{r}u\right)$$

defined for *u* ∈ *C*^2^([*a*, *b*]) and satisfying boundary conditions

(B20) $$u\left(a\right)=0,$$

(B21) $$u^{'}\left(b\right)+\gamma_{k}\left(\lambda ,\right)u\left(b\right)=0.$$

Where *γ*_*k*_(*λ*) is the function given by

(B22) $$\gamma_{k}\left(\lambda \right)=\left\{\begin{array}{cc} -\lambda I_{k}\left(\mathrm{\lambda b}\right)/I_{k}\left(\mathrm{\lambda b}\right) & \lambda \neq 0\\ -k/b & \lambda =0 \end{array}\right),$$

with *I*_*k*_(∙) being the modified Bessel function of the first class and of order *k* for *k* = 0, 1 …, and *λ* ∈ *R*. Notice also that the operator $L_{\lambda }^{k}\left(u\right)$ given by equation (B19), as well as, the boundary conditions (B20) and (B21) originate when eigenfunctions *ϕ*_*λ*_(*r*, *θ*) for the operator *A*_*λ*_ = - ∇^2^ are supposed to have the form *ϕ*_*λ*_(*r*, *θ*) = *R*_*λ*_(*r*)*ψ*(*θ*). The function *γ*_*k*_(*λ*) given by equation (B22) is motivated by the requirement of equation (B15) of radial symmetry in the limiting case *λ* → 0 for *D* > 0 fixed.

For the operator $L_{\lambda }^{k}\left(u\right)$ a Green’s function $H_{\lambda }^{k}\left(r,t\right)$ exists. It is given by

(B23) $$H_{\lambda }^{k}\left(r,t\right)=\left\{\begin{array}{cc} \frac{\left[I_{k}\left(\mathrm{\lambda a}\right)K_{k}\left(\mathrm{\lambda r}\right)-K_{k}\left(\mathrm{\lambda a}\right)I_{k}\left(\mathrm{\lambda r}\right)\right]I_{k}\left(\mathrm{\lambda t}\right)}{I_{k}\left(\mathrm{\lambda a}\right)} & r\leq t\\ \frac{\left[I_{k}\left(\mathrm{\lambda a}\right)K_{k}\left(\mathrm{\lambda t}\right)-K_{k}\left(\mathrm{\lambda a}\right)I_{k}\left(\mathrm{\lambda t}\right)\right]I_{k}\left(\mathrm{\lambda r}\right)}{I_{k}\left(\mathrm{\lambda a}\right)} & t\leq r \end{array}\right),$$

where *K*_*k*_(∙) stands for the modified Bessel function of the second kind and of order *r*. Using formulas (9.6.10) and (9.6.53) in Abranowitz and Stegun [63] we can obtain an expression for $H_{\lambda }^{k}\left(r,t\right)$ depending only on *I*_*k*_(*z*). This permits the proof of the existence of the solution *G*_*λmα*_(*r*, *θ*) in form given by equation (B16) and which depends continuously on the boundary data, namely

(B24) $$C_{\mathrm{\lambda m\alpha}}\left(r,\theta \right)=\sum_{k=0}^{\infty }g_{\mathrm{\lambda m\alpha}}^{k}\left(r\right)e^{\mathrm{\lambda r}\cos\theta }\cos\mathrm{k\theta}$$

with $g_{\mathrm{\lambda m\alpha}}^{k}\left(r\right)$ expressed in terms of the Green’s function $H_{\lambda }^{k}\left(r,t\right)$ through the expression

(B25) $$g_{\mathrm{\lambda m\alpha}}^{k}\left(r\right)=\frac{\sigma_{k}}{D}\int_{a}^{b}\left(-tH_{\lambda }^{k}\left(r,t\right)\left[\int_{-\pi }^{\pi }S^{\mathrm{r\theta}}\left(c,p,q,m,\alpha \right)e^{-\mathrm{\lambda t}\cos\theta }\cos\mathrm{k\theta d\theta}\right]\;\right)\mathrm{dt},$$

and *σ*_*k*_ given by equation (B18) [61].

The symbol *k*_*λmα* +_ denotes the pertinent forward rate constant, which can be obtained by calculating the number of receptors hitting the trap in unit time and dividing it by the average concentration (cf. Eq. (1)). We have nevertheless assumed the existence of a steady-state concentration *C*_*λmα*_(*r*, *θ*) in the reference annulus $\bar{\Omega }$ for all λ. So the number of receptors hitting the trap in unit time coincides with the number of particles that are inserted into the reference annulus in unit time. Hence, we have equivalently

(B26) $$k_{\mathrm{\lambda m\alpha}+}=\frac{\int_{a}^{b}\int_{-\pi }^{\pi }S^{\mathrm{r\theta}}\left(c,p,q,m,\alpha \right)\mathrm{rdrd\theta}}{\frac{1}{\pi b^{2}}\int_{a}^{b}\int_{-\pi }^{\pi }C_{\mathrm{\lambda m\alpha}}\left(r,\theta \right)\mathrm{rdrd\theta}},$$

and by virtue of equations (1) and (3) we must have for *τ*_*λmα*_ the associated mean capture time,

(B27) $$\tau_{\mathrm{\lambda m\alpha}}=\frac{\sum_{k=0}^{\infty }\int_{a}^{b}\int_{-\pi }^{\pi }g_{\mathrm{\lambda m\alpha}}^{k}\left(r\right)e^{\mathrm{\lambda r}\cos\theta }\cos\mathrm{k\theta rdrd\theta}}{\int_{a}^{b}\int_{-\pi }^{\pi }S^{\mathrm{r\theta}}\left(c,p,q,m,\alpha \right)\mathrm{rdrd\theta}}$$

For the case of a uniform receptor insertion (i.e. the case *m* = *b*/*a* and *α* = 0 in equation (B5)), we will have *S*^*rθ*^(*c*, *p*, *q*, *m*, *α*) = *S*, a constant for *a* ≤ *r* ≤ *b*. Denoting by $g_{\mathrm{\lambda s}}^{k}\left(r\right)$ the corresponding form of $g_{\mathrm{\lambda m\alpha}}^{k}\left(r\right)$ and then using the result,

(B28) $$\int_{-\pi }^{\pi }e^{\mathrm{\lambda t}\cos\theta }\cos\mathrm{k\theta d\theta}=2\pi ∙{\left(-1\right)}^{k}I_{k}\left(\mathrm{\lambda t}\right)$$

(cf. Abramowitz and Stegun [63]) equation (B25) yields

(B29) $$g_{\mathrm{\lambda S}}^{k}=\frac{2\mathrm{\pi S}\sigma_{k}{\left(-1\right)}^{k+1}}{D}\int_{a}^{b}tH_{\lambda }^{k}\left(r,t\right)I_{k}\left(\mathrm{\lambda t}\right)\mathrm{dt}.$$

Meanwhile, the corresponding mean capture time *τ*_*λs*_ becomes

(B30) $$\tau_{\mathrm{\lambda s}}=\frac{\sum_{k=0}^{\infty }\vartheta_{k}{\left(-1\right)}^{k+1}\int_{a}^{b}r\left\{\int_{a}^{b}tH_{\lambda }^{k}\left(r,t\right)I_{k}\left(\mathrm{\lambda t}\right)\mathrm{dt}\right\}I_{k}\left(\mathrm{\lambda r}\right)\mathrm{dr}}{D\left(b^{2}-a^{2}\right)},$$

where

$$\vartheta_{k}=\left\{\begin{array}{ccc} 2 & \mathrm{if} & k=0\\ 4 & \mathrm{if} & k\geq 1 \end{array}\right).$$

It can be also shown [61] that for *D* > 0 and fixed, we have

(B31) $$\lim_{{}_{\lambda \to \infty }}\tau_{\mathrm{\lambda s}}=\frac{b^{4}\ln\left(\frac{b}{a}\right)}{2D\left(b^{2}-a^{2}\right)}-\frac{3b^{2}-a^{2}}{8D};$$

that is, if receptor insertion is uniform all over *Ω*, then whenever *λ* approaches zero, *τ*_*λs*_ approaches the value *τ*_*du*_ of equation (12), calculated by Berg and Purcell [23].

## Competing interests

The authors declare that they have no competing interests.

## Authors’ contributions

HEH conceived, designed and performed the research. CLR performed the required mathematical proofs and numerical analysis procedures. OC supervised the research and revised the manuscript critically at a formal level. All authors read and approved the final manuscript.
